# Role of Exercise in Cardiovascular Disease and Alzheimer’s Disease Comorbidity: A Blood Biomarker Perspective

**DOI:** 10.3390/cells15141284

**Published:** 2026-07-17

**Authors:** Yu Lu, Chunyan Xu, Pengyu Fu, Lijing Gong

**Affiliations:** 1 School of Sport Science, Beijing Sport University, Beijing 100084, China; 2023011848@bsu.edu.cn; 2 Key Laboratory of Physical Fitness and Exercise, Ministry of Education, Beijing Sport University, Beijing 100084, China; 3 Beijing Sports Nutrition Engineering Research Center, Beijing 100084, China; 4 Department of Physical Education, Northwestern Polytechnical University, Xi’an 710072, China; fupy@nwpu.edu.cn; 5 China Institute of Sport and Health Science, Beijing Sport University, Beijing 100084, China; 6 Key Laboratory for Performance Training & Recovery of General Administration of Sport, Beijing 100084, China

**Keywords:** cardiovascular disease, Alzheimer’s disease, comorbidity, blood biomarkers, exercise

## Abstract

**Highlights:**

**What are the main findings?**

- Exercise acts as a multi-target intervention that disrupts the CVD-AD pathological cascade by improving cerebral perfusion, clearing Aβ, and preserving blood–brain barrier integrity.
- Blood biomarkers (p-tau217, NT-proBNP, GFAP, NfL, and miRNAs) exhibit distinct stage-specific profiles during comorbidity progression, which can be dynamically modulated by multi-target exercise interventions.

**What are the implications of the main findings?**

- A systematic analysis of these blood biomarkers is expected to provide a valuable translational medical perspective for minimally invasive clinical monitoring of disease progression and for evaluating the potential clinical benefits of different forms of exercise.
- Highlighting the current clinical data gap regarding exercise-drug-nutrition interactions provides a crucial baseline for utilizing AI and multi-omics to advance precise, personalized comorbidity management.

**Abstract:**

With the growing of the global population aging, the comorbidity of cardiovascular disease (CVD) and Alzheimer’s disease (AD) has gradually become a major contributor to global disease burden, with the two diseases exhibiting interacting pathological characteristics. This review summarizes the mechanisms underlying CVD-AD comorbidity, including cerebral hypoperfusion and protein aggregation, oxidative stress and inflammation, as well as metabolic disorders and genetic factors. We also assess the value of blood biomarkers in this comorbidity, such as p-tau217, NT-proBNP, NfL, GFAP, and miRNAs. We then review the role of exercise in ameliorating this comorbidity, integrating its potential mechanisms into five aspects: repairing cerebral hypoperfusion and endothelial injury, accelerating Aβ clearance and inhibiting Tau protein hyperphosphorylation, reducing inflammation, protecting neuronal structure and the blood–brain barrier, and improving metabolic disorder. We also analyze in detail the relationship between exercise targets and blood biomarkers. Finally, we discussed the impact and application of different exercise modalities in this comorbidity, concluding with suitable exercise prescriptions and relevant safety considerations for each modality, further organized the mainstream pharmacological and nutritional intervention strategies for CVD-AD comorbidity, and objectively explored their potential interactions when combined with exercise interventions. Future research should integrate multi-omics technologies and gradually refine clinical data on comorbidity, with the aim of developing more targeted, personalized exercise prescriptions for patients with comorbidities, thereby providing scientific theoretical guidance for early risk prevention and disease management in patients with co-occurring CVD and AD.

## 1. Introduction

Cardiovascular disease (CVD) and Alzheimer’s disease (AD), two major causes of disability and death among the elderly, have been the focus of extensive research. According to a World Health Organization report, CVD remains the leading cause of death worldwide, involving heart and vascular disorders like atherosclerosis (AS), heart failure (HF), atrial fibrillation (AF), and coronary artery disease (CAD). Meanwhile, the global prevalence of dementia continues to rise. As a neurodegenerative disease, AD is one of the most common types of dementia, characterized by senile plaques formed by amyloid-β (Aβ) deposits, neurofibrillary tangles (NFTs) formed by hyperphosphorylated Tau protein, and neuronal damage [1,2]. CVD and AD exhibit a strong comorbidity association. CVD is a major risk factor for AD, and this link is supported by both observational studies and experimental data. Cardiovascular health studies have shown that older adults with CVD (excluding stroke) have a higher risk of dementia and AD than their counterparts without CVD [3]. Similarly, studies have demonstrated that one-third of AD-related dementia cases are attributable to modifiable atherosclerotic cardiovascular risk factors [4]. Conversely, the brain can also modulate cardiac function; autonomic dysfunction resulting from neurodegenerative diseases may lead to arrhythmia, thus triggering the onset of CVD [5].

Although the epidemiological link between CVD and AD is well established, the mechanisms underlying their comorbidity are complex. From macro-level cerebral hypoperfusion to micro-level oxidative stress and inflammation, and further to metabolic disorders and shared genetic risk factors, the brain and heart are interconnected and interact through the heart-brain axis. Both CVD and AD are progressive diseases. In current clinical practice, when patients with cardiovascular risk factors present with cognitive decline, the cardiovascular system has already been causing injury to brain tissue for decades [6]. In this context, identifying early diagnostic clinical indicators for this comorbidity is particularly important. Neuroimaging techniques for detecting brain atrophy and protein deposition are limited in their large-scale application to cardiovascular risk populations due to high cost. Blood biomarkers such as phosphorylated tau at threonine 217 (p-tau217), N-terminal pro-B-type natriuretic peptide (NT-proBNP), Neurofilament light chain (NfL), Glial fibrillary acidic protein (GFAP), and MicroRNAs (miRNAs) also serve as indicators for monitoring and preventing comorbidity, playing a key role in CVD-AD comorbidity.

Currently, interventions for CVD-AD comorbidity mainly include pharmacotherapy and nutritional intervention. However, sole reliance on pharmacotherapy and nutritional supplementation has certain limitations, including potential side effects and long-term high costs, making it difficult to establish sustainable intervention strategies. Exercise, as a multi-target non-pharmacological intervention, can rebuild the brain-heart connection and ameliorate the comorbidity of CVD and AD. Based on this, the review aims to delineate the comorbidity mechanisms of CVD and AD, summarize potential blood biomarkers, and explore the physiological mechanism by which exercise improves this comorbidity. We will compare the effects of different exercise modalities based on existing evidence, and provide exercise prescription recommendations to support early management and intervention for this comorbidity.

## 2. Search Strategy and Selection Criteria

This narrative review is based on a comprehensive literature search of PubMed, Web of Science, and Embase covering the past decade. Given the interdisciplinary nature of the comorbidity of CVD and AD, this study employed a modular search strategy. The literature search focused on four core thematic areas: (1) comorbidity of CVD and AD, with primary keywords: “Cardiovascular Disease”, “Hypertension”, “Heart Failure”, “Atrial Fibrillation”, “Atherosclerosis”, “Alzheimer’s Disease”, “Dementia”, “Cognitive Impairment”, “Comorbidity”, “Co-occurrence”, “Multimorbidity”, “Heart-brain axis”, “Shared pathophysiology”, “Risk factors”. (2) blood biomarkers for cardiovascular disease and Alzheimer’s disease, with primary keywords: “Blood-based biomarkers”, “Plasma biomarkers”, “Amyloid-beta”, “Tau protein”, “Phosphorylated tau”, “NT-proBNP”, “BNP”, “NfL”, “GFAP”, “miRNAs”. (3) exercise and physical interventions, with primary keywords: “Exercise intervention”, “Physical activity”, “Aerobic exercise”, “Resistance training”, “Strength training”, “High-intensity interval training”, “Combined exercise”, “Tai Chi”, “Yoga”. (4) adjunctive interventions (pharmacotherapy and nutrition), with primary keywords: “Pharmacotherapy”, “Drug treatment”, “Nutritional intervention”, “Dietary patterns”, “Mediterranean diet”. Only peer-reviewed articles published in English were selected for screening and inclusion.

## 3. Pathogenic Mechanisms of CVD and AD Comorbidity

### 3.1. Cerebral Hypoperfusion and Protein Aggregation

Cerebral hypoperfusion characterized by decreased cerebral blood flow, further drives cerebral ischemic-hypoxic injury. It is a common mechanism underlying the pathogenesis of AD and also links the pathological progression of CVD and AD. The causes of cerebral hypoperfusion can be divided into three main categories: structural lesions resulting from arterial stenosis or occlusion, changes in cerebrovascular hemodynamics, and increased blood viscosity due to alterations in blood composition [7]. These mechanisms are closely associated with cardiovascular diseases that reduce cerebral blood flow, such as AS, HF, AF. In patients with AS, the proximal arterial vessels cannot effectively absorb blood flow pressure. This pressure then impacts the vulnerable distal cerebral microcirculation, leading to microvascular injury and reduced cerebral perfusion reserve, thereby accelerating the progression of CVD and AD comorbidity [8,9]. Patients with HF experience long-term chronic cerebral hypoperfusion due to low cardiac output, which increases the risk of AD. Studies indicate that 40% to 60% of patients with HF exhibit some degree of cognitive impairment [10]. Furthermore, AF is also a strong risk factor for AD. Experimental evidence shows that AF patients have cerebral hypoperfusion. Moreover, AF patients with concomitant HF show slower cerebral blood flow and more severe cognitive impairment [11].

Both AS, HF and AF can cause the cerebral microcirculation to remain in a state of chronic hypoxia. Hypoxia from cerebral hypoperfusion activates hypoxia-inducible factor-1α (HIF-1α), which enhances the expression of β-secretase (BACE1) and γ-secretase via transcriptional and non-transcriptional pathways, respectively. BACE1 and γ-secretase then sequentially cleave the amyloid precursor protein (APP) to generate Aβ [12,13,14]. HIF-1α-mediated Aβ production is an adaptive response to hypoxic–ischemic injury aimed at protecting neurons. However, under long-term low-perfusion conditions, this response leads to blood–brain barrier (BBB) dysfunction, preventing Aβ clearance through normal transport and metabolic pathways [9]. Consequently, Aβ accumulates massively within brain parenchyma and vascular walls, exacerbating the comorbidity of CVD and AD.

### 3.2. Oxidative Stress and Inflammation

Oxidative stress is essentially an imbalance between the production of reactive oxygen species (ROS) and the capacity of the endogenous antioxidant system [10]. In the cardiovascular and nervous systems, physiological concentrations of ROS act as signaling molecules to maintain homeostasis. However, under pathological conditions, excessive ROS activates the transcription factor NF-κB and induces tumor necrosis factor-α (TNF-α), interleukin-1β (IL-1β), and interleukin-6 (IL-6), leading to a transition from oxidative stress to chronic low-grade inflammation and affecting the heart-brain axis [15,16,17].

Mitochondria are the primary sites of ROS production in the brain and heart; they are also key targets of oxidative damage. Studies have shown that when Aβ toxicity or vascular stress causes increased mitochondrial permeability, the pathological mitochondrial permeability transition pore (mPTP) opens. At the same time, mitochondrial DNA (mtDNA) is released into the cytoplasm, where it activates the cGAS-STING signaling or directly activates the NLRP3 inflammasome, concurrently triggering immune dysregulation in the heart and brain tissues [9,15,18]. The accumulation of misfolded proteins (such as Aβ oligomers and Tau) can induce serious endoplasmic reticulum (ER) stress and activate the unfolded protein response (UPR). If this stress persists, it will drive caspase-12-mediated apoptosis through the upregulation of ATF4 and CCAAT/enhancer-binding protein homologous protein (CHOP), thereby accelerating neuronal loss and cardiac dysfunction [19]. A recent study on the “neuro-cardiac inflammasome axis” found that the brains of patients with AD produce extracellular vesicles (EVs). These EVs carry inflammatory factors like caspase-1 and IL-1β, can cross the BBB to enter the systemic circulation, and subsequently cause peripheral vascular and heart inflammation [20]. Furthermore, under inflammatory stress, asparagine endopeptidase (AEP) cleaves APP and Tau via the C/EBPβ/AEP signaling pathway, simultaneously exacerbating brain neurodegeneration and carotid plaque [21]. Therefore, ROS dysregulation, mitochondrial damage, proteotoxic stress, as well as the crosstalk-driven interplay between oxidative stress and chronic inflammation constitute the core mechanisms underlying CVD-AD comorbidity.

### 3.3. Metabolic Disorders and Genetic Factors

Metabolic disorders drive the comorbidity of CVD and AD, mainly through insulin resistance (IR) and dyslipidemia. IR is not only a typical cardiovascular risk factor but also a key factor linking vascular damage to neurodegeneration. In the peripheral vascular system, IR disrupts PI3K signaling, thereby inhibiting nitric oxide (NO) synthesis in endothelial cells. Concurrently, activation of the MAPK pathway promotes endothelin-1 (ET-1) expression at the vascular level, resulting in sustained vasoconstriction and reduced tissue blood flow, ultimately accelerating the progression of AS and contributing to cerebral hypoperfusion [22]. In the central nervous system, insulin signaling dysfunction increases the activity of glycogen synthase kinase-3β (GSK-3β), thus driving Tau phosphorylation and neuronal tangle formation [23]. Research indicates that insulin-degrading enzyme (IDE) plays a vital role in regulating insulin and Aβ clearance. Although the exact effects of IR on IDE remain unclear, insulin competitively binds to IDE in a hyperinsulinemic environment, which impedes Aβ clearance in the brain and accelerates the pathological accumulation of this toxic protein [19,24].

Previous research has shown that there is a strong shared genetic basis between CVD and AD [25]. At the level of lipid metabolism, the APOE4 genotype is the strongest genetic risk factor for AD and is also a well-known risk factor for CVD. APOE4 can accelerate the production of Aβ in the brain by enhancing BACE1 activity [26]. Meanwhile, in APOE4 carriers, the preference of APOE4 for triglyceride-rich lipoproteins leads to the downregulation of low-density lipoprotein (LDL) receptors in hepatocytes, resulting in reduced efficiency of LDL uptake and elevated plasma LDL levels, which promote the development of AS [19,27]. Similarly, as a multifunctional regulator, the DAB2IP gene also plays a crucial role in both the cardiovascular and nervous systems. On the one hand, the absence of DAB2IP leads to impaired lipid clearance, accelerating the progression of AS plaques; on the other hand, DAB2IP deficiency results in sustained activation of apoptotic pathways, causing damage to hippocampal and cortical neurons as well as BBB structure, and triggering a series of neurodegenerative lesions [28]. Furthermore, at the immune-inflammatory level, Wang et al. identified the WIPF3 gene as a gene shared by CVD and AD through bioinformatics analysis. In AD, downregulation of WIPF3 promotes microglial-mediated neuroinflammation and accelerates neuronal damage; in AS, insufficient WIPF3 expression promotes the infiltration of immune cells into plaques and enhances inflammatory responses in the vascular wall [29]. Thus, metabolic disorders—by increasing Aβ aggregation, impairing vascular function, and disrupting cellular signaling—together with relevant genetic risk factors, constitute key pathological links in the comorbidity of CVD and AD.

**Figure 1:**
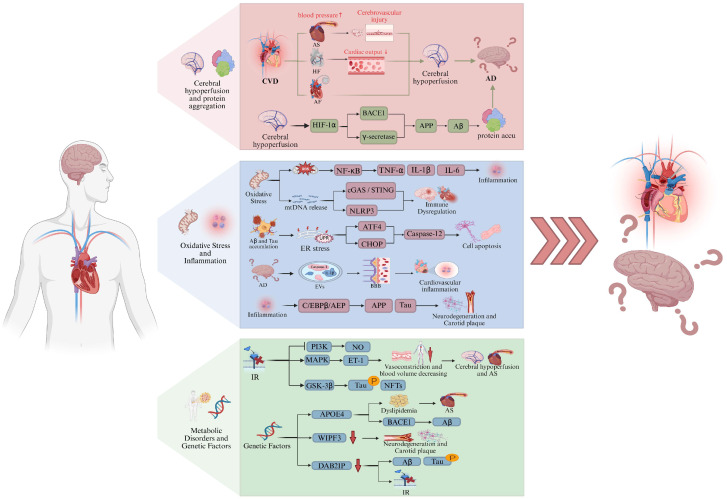
The possible pathogenic mechanisms of CVD and AD comorbidity. ⟶: This symbol represents a promoting effect. ⟞: This symbol represents an inhibitory effect. Abbreviations: AD, Alzheimer’s disease; AS, atherosclerosis; AF, atrial fibrillation; Aβ, amyloid-β; APP, amyloid precursor protein; ATF4, activating transcription factor 4; AEP, asparagine endopeptidase; APOE, apolipoprotein E; BACE1, β-secretase; BBB, blood–brain barrier; CVD, cardiovascular disease; CHOP, CCAAT/enhancer-binding protein homologous protein; C/EBPβ, CCAAT/enhancer-binding protein β; DAB2IP, DAB2 interacting protein; ER, endoplasmic reticulum; EVs, extracellular vesicles; ET-1, endothelin-1; GSK-3β, glycogen synthase kinase-3β; HF, heart failure; HIF-1α, hypoxia-inducible factor-1α; IL-1β, interleukin-1β; IL-6, interleukin-6; IR, insulin resistance; mtDNA, mitochondrial DNA; NF-κB, nuclear factor kappa-B; NO, nitric oxide; NFTs, neurofibrillary tangles; PI3K, phosphoinositide 3-kinase; ROS, reactive oxygen species; TNF-α, tumor necrosis factor-α; UPR, unfolded protein response; WIPF3, WAS/WASL interacting protein family member 3. Created in BioRender. Lu, Y. (2026) https://BioRender.com/tw0pzyr (accessed on 15 July 2026).

The mechanisms linking cerebral hypoperfusion and protein aggregation, oxidative stress and inflammation, and metabolic disorders with genetic factors in CVD-AD comorbidity are summarized in Figure 1.

## 4. Blood Biomarkers for CVD and AD Comorbidity

The biomarkers for AD can be detected not only in cerebrospinal fluid (CSF) but also in other biological matrices such as plasma and saliva [30]. Among these, plasma biomarker testing is an ideal diagnostic option, as it is less invasive, more accessible, and offers reliable detection performance. The latest revisions to the AD diagnostic criteria by the National Institute on Aging (NIA) and the Alzheimer’s Association (AA) have incorporated blood biomarkers and introduced the expanded ATX(N) model, which stands for amyloid (A), tau (T), neurodegeneration (N), and emerging biomarkers (X) that capture additional pathological processes (such as neuroinflammation, synaptic dysfunction, and blood–brain barrier damage) [31]. Similarly, we classify blood biomarkers for CVD-AD comorbidity into T (p-tau217), N (NfL), X (GFAP), as well as NT-proBNP closely associated with CVD and miRNAs associated with comorbidity.

### 4.1. p-tau217

The primary physiological function of Tau protein is to bind to and stabilize neuronal microtubules. In the pathological progression of AD, Aβ plaque deposition disrupts the balance between kinase activation and phosphatase inhibition, leading to excessive phosphorylation of Tau protein at multiple sites, including Thr181, Thr217, and Thr231 [32]. A recent study has shown that plasma levels of the AD-associated biomarker p-tau correlate well with CSF levels, and can serve as surrogates of the corresponding cerebrospinal fluid biomarker [30]. However, different phosphorylation sites may have different diagnostic value; plasma p-tau217 is currently recognized as one of the most promising biomarkers, with studies indicating that it significantly outperforms traditional p-tau181 [33]. It should be noted that there are significant differences in the concentrations of Aβ42, Aβ40, and p-tau across different biological matrices such as CSF, plasma, and saliva [30]. Therefore, future studies need to establish a plasma-specific reference range for p-tau217. For patients with CVD, plasma p-tau217 demonstrates a unique clinical predictive role. In a cohort study involving 162 asymptomatic adults with CVD, approximately 55% of participants exhibited elevated plasma p-tau217 levels, far higher than those of a general control population with similar age and genetic background (22%). Further analysis showed that plasma p-tau217 levels were significantly negatively correlated with Montreal Cognitive Assessment (MoCA) scores, and this association persisted even after adjusting for a history of stroke. These findings suggest that plasma p-tau217 can identify CVD patients who have not yet met the diagnostic criteria for dementia but already exhibit early AD pathology [34]. Similarly, a longitudinal study of older adults with CVD found that individuals with positive Tau markers decline significantly faster in domains such as episodic memory and executive function than other groups. These findings further support the role of p-tau217 in predicting future cognitive decline in CVD patients [35]. Furthermore, baseline plasma p-tau217 levels are significantly elevated in patients with comorbidity. In cognitively impaired individuals with a history of CVD or impaired renal function, plasma p-tau217 concentration typically increases by 1.1 to 1.3 times [33]. Therefore, plasma p-tau217 can serve as a blood biomarker for the early identification of patients with CVD and AD comorbidity.

### 4.2. NT-proBNP

NT-proBNP is an endocrine signal produced by the ventricular wall in response to excessive pressure or volume load [36]. In the subclinical stage, high NT-proBNP and brain natriuretic peptide (BNP) levels in plasma indicate cardiac dysfunction, such as reduced pumping efficiency and decreased cardiac output [37]. Studies have shown that endothelial damage and cerebral microvascular sparsity resulting from aortic stiffness are factors promoting the comorbidity of CVD and AD. This process is often accompanied by an elevation of NT-proBNP levels in plasma [38,39]. Moreover, elevated plasma NT-proBNP levels can independently predict AD and are strongly associated with cognitive decline. Both cross-sectional and longitudinal studies indicate that high plasma NT-proBNP levels are linked to decreased processing speed and memory, white matter injury, brain atrophy, and silent cerebral infarction, while also suggesting cerebral hypoperfusion and microvascular pathology [40]. In another cohort study, this damage was quantified: for each standard deviation increase in plasma NT-proBNP, the resulting degree of brain degeneration was equal to an accumulated loss of 1.2 years of gray matter volume, an accumulated increase of 1.4 years in white matter lesion burden, and an acceleration in the rate of white matter fiber degeneration by nearly 4 years [39]. Similarly, longitudinal data have established the central role of plasma NT-proBNP in predicting future dementia risk; that is for every 1 log unit increase in NT-proBNP, the risk of dementia increases by 21–28% [41]. In clinical practice, even in individuals without obvious HF and NT-proBNP within the normal range, elevated levels are markedly related to poorer overall cognition, processing speed, and executive function [42]. This demonstrates the potential of plasma NT-proBNP as a biomarker for the early identification of heart and brain impairments. In summary, plasma NT-proBNP is not only an important cardiac biomarker but also a predictor of AD and other types of dementia.

### 4.3. NfL

NfL, a key component of the neuronal cytoskeleton, is abundantly concentrated in neurons with myelinated axons, and its elevated levels (in plasma) serve as a universal marker of axonal damage [43]. In CVD-AD comorbidity, particularly in the later stages of the disease, plasma NfL (pNfL) is one of the classic indicators for assessing the extent of structural brain damage [44,45]. Under conditions of vascular leakage or endothelial dysfunction, abnormal elevation of placental growth factor leads to an increase in white matter hyperintensity (WMH) volume, subsequently inducing glial neuroinflammation and Tau protein phosphorylation, resulting in the loss of neuronal axonal integrity and releasing NfL into the circulatory system [46]. pNfL occupies a downstream position in this neurovascular axis pathological cascade, reflecting the final state of nerve fiber damage. In the Multiethnic Study of Atherosclerosis (MESA) cohort, pNfL levels showed a strong positive association with cerebrovascular microstructural damage, indicating its potential value in quantifying the extent of vasculogenic brain injury [47]. pNfL dynamically responds to an individual’s overall cardiovascular health status. A Chicago Health and Aging Project cohort study demonstrated that individuals with higher cardiovascular health scores had markedly lower baseline pNfL concentrations and a slower annual rate of increase with age [48]. This suggests that promoting cardiovascular health may reduce the burden of neurodegenerative diseases in older adults, and that pNfL can serve as a key biomarker for evaluating the efficacy of cardiovascular health interventions. In the background of CVD-AD comorbidity, although pNfL is elevated in various neurological diseases and its specificity for diagnosing AD is inferior to that of plasma p-tau217, it holds significant value in assessing disease progression rates, preventing cognitive decline caused by comorbidity, and evaluating patients’ responsiveness to therapeutic interventions [49,50].

### 4.4. GFAP

GFAP is an intermediate protein specific to astrocytes [51]. It participates in cytoskeletal assembly and morphological maintenance, and its upregulation reflects a state of glial cell activation [52]. In current comorbidity studies, GFAP serves as a key mediator linking vascular injury to early inflammation in AD [46,47,52]. It has been reported that high GFAP expression is often triggered by vascular stress. Elevated pro-angiogenic factors increase WMH volume, which induces astrocyte activation and GFAP release, thereby promoting neuroinflammation [46]. Importantly, this astrocytic inflammatory state is not merely a byproduct of vascular injury but a key driver of the core pathological progression of AD. Experimental evidence indicates that the presence of Aβ alone is incapable of driving large-scale Tau protein spread. Only when GFAP levels are raised can Aβ deposits significantly accelerate Tau phosphorylation and spread throughout the brain [53]. This suggests that Aβ-induced Tau pathological progression shows marked GFAP dependence. Additionally, the MESA cohort demonstrated a positive association between GFAP and increased WMH volume as well as hippocampal atrophy, highlighting its unique value in assessing the damage caused by vascular inflammation to brain structural integrity [47]. Plasma GFAP is a sensitive biomarker of early AD pathology, particularly in the preclinical stage where intracerebral Aβ deposition has already occurred but significant cognitive impairment has yet to manifest; its expression increases earlier than axonal injury markers such as pNfL [54,55]. Therefore, as a biochemical indicator for assessing neuroinflammation, plasma GFAP can sensitively capture the turning point at which CVD risk transitions to central nervous system inflammation. Combined detection of plasma GFAP and p-tau217 can more accurately predict decline across the entire cognitive domain and holds potential for optimizing the stratification of mixed AD pathologies [56,57].

### 4.5. miRNAs

miRNAs are a class of small non-coding RNAs. Their primary function is to regulate protein levels by modulating mRNA degradation, thereby influencing cell proliferation, differentiation, apoptosis, metabolism, and immune responses [58]. miRNAs can be used not just for AD diagnosis and prognosis, but also as a faster and more accurate tool for diagnosing acute CVD or HF [59,60,61]. In the context of comorbidity, damaged cardiovascular endothelial or myocardial cells release EVs containing specific miRNAs into the circulatory system. Because these miRNAs are packaged within lipid vesicles or protein complexes, they exhibit high stability in blood and cerebrospinal fluid, enabling them to cross the BBB as signaling molecules and transmit cardiovascular stress signals from the peripheral circulatory system to the central nervous system, thereby inducing brain damage such as AD [39,58,62]. In lipid metabolism and amyloid pathology, miR-33 is a key regulator of cholesterol homeostasis. By inhibiting ATP-binding cassette subfamily A member 1 to reduce high-density lipoprotein (HDL) production. Overexpression of miR-33 not only worsens AS but also reduces Aβ clearance efficiency, thereby promoting amyloid plaque deposition. In addition, miR-425 plays a key role in endothelial senescence and neurodegeneration. It is significantly upregulated in high-risk cardiovascular conditions like obesity and diabetes, and also drives vascular endothelial cell apoptosis, abnormal Tau protein phosphorylation, and neuronal apoptosis [62]. Under chronic inflammation, the pro-inflammatory factor TNF-α induces the overexpression of miR-146a in the brains of AD patients, triggering neurovascular inflammation and resulting in secondary neuronal damage [63]. Other miRNAs exhibit downregulation or deficiency under pathological conditions. For example, downregulation of miR-126a-5p, miR-532-5p, and miR-222 is associated with heart failure, neurological deficits, and cardiac hypertrophy, respectively [64,65,66]. This evidence indicates that abnormal miRNA expression is not only associated with vascular endothelial injury, Aβ deposition in AD, Tau pathology, and synaptic dysfunction, but also shows biomarker potential in reflecting systemic metabolic disorder and complex cross-organ regulatory networks.

## 5. Protective Mechanisms of Exercise Against CVD and AD Comorbidity

In the comorbidity of CVD and AD, exercise serves as a classic non-pharmacological intervention that mainly slows the pathological process by improving hemodynamics, maintaining protein homeostasis, reducing neuroinflammation, protecting neural integrity, and improving metabolic disorders. Exercise is not only an effective way to maintain cardiovascular health and cognitive function, but also regulates molecular signaling pathways to influence the heart-brain axis, thereby providing direct or indirect protection to the cardiovascular system and the brain.

### 5.1. Exercise Repairs Cerebral Hypoperfusion and Endothelial Injury

Persistently elevated levels of BNP and NT-proBNP are important markers for assessing HF and are also associated with subclinical brain injury and cognitive impairment. The effects of exercise on plasma BNP and plasma NT-proBNP occur in two phases: short-term and long-term. An animal study showed that short-term high-intensity exercise upregulates the expression of the myocardial functional receptor NPR-A and downregulates the NPR-C receptor, thereby increasing BNP secretion. BNP binds to the NPR-A receptor, activating the BNP/cGMP signaling pathway to reduce peripheral resistance and cardiac workload, thus improving myocardial perfusion [67]. A clinical trial has shown that long-term, regular aerobic exercise enhances patients’ cardiopulmonary function, improves ventricular structure, reduces ventricular wall stress, and decreases the need for BNP secretion. Consequently, it lowers baseline levels of BNP and NT-proBNP, improves cardiac pumping function and alleviates cerebral hypoperfusion associated with comorbidities [68]. Findings indicate that among HF patients undergoing exercise rehabilitation, those with a greater post-exercise increase in NT-proBNP have a 1.8-fold higher risk of mortality compared to those with a smaller increase, and this result is independent of other factors such as age and left ventricular ejection fraction [69]. This suggests that changes in the increase in NT-proBNP levels can be used to monitor the effectiveness of exercise interventions during patient rehabilitation, while also serving as a warning of poor prognostic risk in patients with persistently elevated post-exercise NT-proBNP levels.

In addition to improving cardiac function and low perfusion by regulating BNP and NT-proBNP, exercise can also influence miRNA levels to improve systemic hemodynamics and repair endothelial injury. miR-126 is a miRNA enriched in endothelial cells and endothelial progenitor cells. It regulates angiogenesis, inhibits inflammation and apoptosis, acts as a cardioprotective factor, and exhibits reduced levels in the pathophysiology of CVD-AD comorbidity [70,71]. Exercise training can upregulate miR-126 expression in obese individuals and rats. Further studies have shown that exercise downregulates phosphoinositide-3-kinase regulatory subunit 2 (PIK3R2) and sprout-related evh1 domain-containing 1 (SPRED1) in rats with high miR-126 expression, subsequently activating the PI3K/AKT/eNOS and MAPK signaling pathways to promote angiogenesis, repair endothelial cells, and improve cardiac function [72,73]. Exercise-mediated downregulation of PIK3R2 and SPRED1 also promotes vascular endothelial growth factor (VEGF) signaling, regulates and restores the integrity of cerebral microvasculature and cardiovascular systems, improving the function of the cardio-cerebral axis [74]. Furthermore, a preclinical study showed that exercise induces physiological cardiac hypertrophy by increasing miR-222 to suppress HMBOX1 gene expression. The upregulation of miR-222 also inhibits p27 and homeodomain-interacting protein kinase 1 (HIPK1), promoting cardiomyocyte proliferation, thus improving cerebral hypoperfusion and cellular injury in the context of CVD-AD comorbidity [75,76].

**Figure 2:**
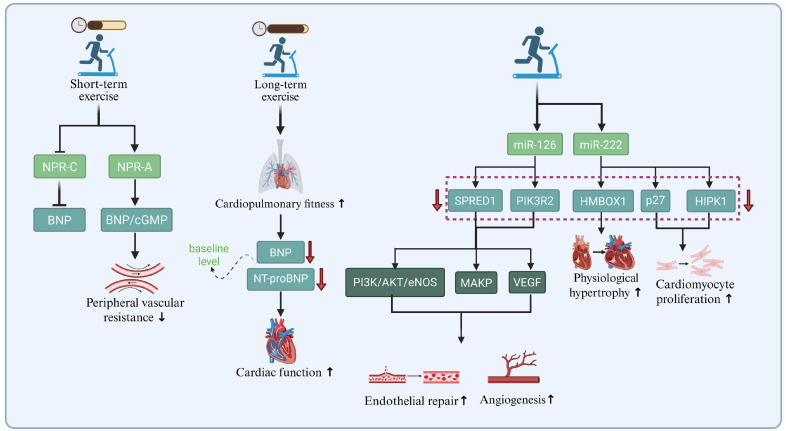
The mechanisms about exercise repairs cerebral hypoperfusion and endothelial injury in CVD-AD comorbidity. ⟶: This symbol represents a promoting effect. ⟞: This symbol represents an inhibitory effect. ↓: This symbol represents a horizontal decline. ↑: This symbol represents a horizontal rise or an enhancement of a function. Abbreviations: AKT, protein kinase B; BNP, brain natriuretic peptide; cGMP, cyclic guanosine monophosphate; eNOS, endothelial nitric oxide synthase; HIPK1, homeodomain-interacting protein kinase 1; HMBOX1, homeobox containing 1; MAPK, mitogen-activated protein kinase; NPR-A, natriuretic peptide receptor A; NPR-C, natriuretic peptide receptor C; NT-proBNP, N-terminal pro-B-type natriuretic peptide; p27, cyclin-dependent kinase inhibitor p27 (or cyclin-dependent kinase inhibitor 1B); PI3K, phosphoinositide 3-kinase; PIK3R2, phosphoinositide-3-kinase regulatory subunit 2; SPRED1, sprouty related evh1 domain containing 1; VEGF, vascular endothelial growth factor. Created in BioRender. Lu, Y. (2026) https://BioRender.com/9ybwyg7 (accessed on 15 July 2026).

The mechanisms about exercise repairs cerebral hypoperfusion and endothelial injury in CVD-AD comorbidity are summarized in Figure 2.

### 5.2. Exercise Accelerates Aβ Clearance and Inhibits Tau Hyperphosphorylation

Aβ deposition and Tau protein hyperphosphorylation are common pathological features of AD and are also closely associated with CVD pathologies like cardiac dysfunction and AS. Based on this, exercise improves CVD-AD comorbidity by clearing Aβ and p-tau, while also inhibiting their production. The clearance of Aβ and abnormal Tau depends on aquaporin channel 4 (AQP4), which is primarily expressed in brain glial cells and epithelial cells of peripheral organs. In AD, AQP4 diffuses from astrocyte dendrites to other locations such as the cell body or processes, exhibiting a depolarized distribution [77]. Evidence from an animal study indicates that high-intensity interval training (HIIT) enhances the polarization of microglia from pro-inflammatory M1 toward anti-inflammatory M2, while driving astrocytes from pro-inflammatory A1 toward neuroprotective A2. This process increases AQP4 expression and restores its polarized distribution [2]. The exercise-mediated polarization enhances the inflow of the cerebral lymphatic system, transporting abnormal Tau protein and Aβ from the cortex and hippocampus into the peripheral circulatory system, finally being cleared through renal excretion. In this way, it reduces the aggregation of Aβ and p-tau, as well as the impairment of synaptic function.

The E3 ubiquitin ligase plays a crucial role in degrading abnormal proteins inside the cell. It includes the carboxyl terminus of HSC70-interacting protein (CHIP) and ubiquitin carboxyl-terminal esterase-1 (UCHL-1). CHIP interacts with heat shock protein 70 (HSP70) to form a CHIP/HSP complex, promoting the ubiquitination of misfolded proteins and delivering target proteins to proteases for degradation [78]. UCHL-1 is involved in the degradation of APP and BACE1, and its levels are downregulated in the early stages of AD [79]. Studies have shown that treadmill exercise can activate the PI3K/Akt pathway in the hippocampus of AD model mice, and increase the transcription and expression of HSC70 and CHIP to reduce the formation of phosphorylated Tau. Additionally, as a downstream product of the PI3K/Akt signaling pathway, UCHL-1 reduces Aβ production by inhibiting the generation of APP and BACE1, thereby slowing the progression of AD [80].

**Figure 3:**
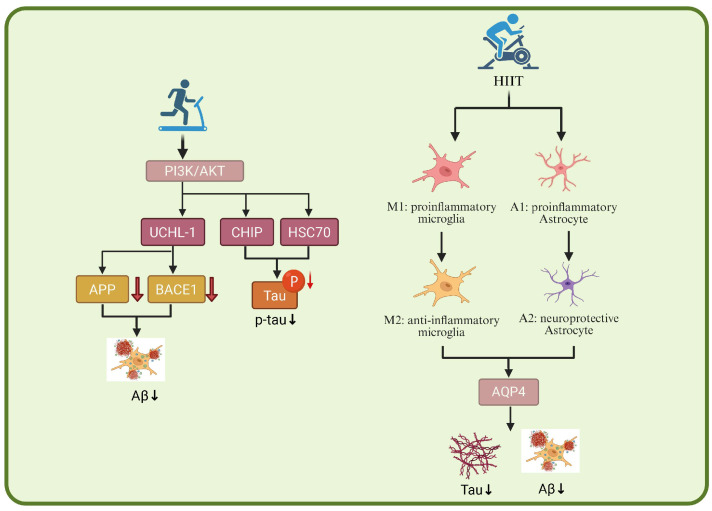
The mechanisms about exercise accelerate Aβ clearance and inhibit tau hyperphosphorylation in CVD-AD comorbidity. ↓: This symbol represents a horizontal decline. Abbreviations: Aβ, amyloid-β; AKT, protein kinase B; APP, amyloid precursor *protein*; AQP4, aquaporin channel 4 (or aquaporin-4); BACE1, β-secretase (beta-site APP cleaving enzyme 1); CHIP, carboxyl terminus of HSC70-interacting protein; HIIT, high-intensity interval training; HSC70, heat shock cognate protein 70; PI3K, phosphoinositide 3-kinase; UCHL-1, ubiquitin C-terminal hydrolase L1. Created in BioRender. Lu, Y. (2026) https://BioRender.com/meqf5ty (accessed on 15 July 2026).

The mechanisms about exercise that accelerate Aβ clearance and inhibit tau hyperphosphorylation in CVD-AD comorbidity are summarized in Figure 3.

### 5.3. Exercise Reduces Inflammation

Primary cilia are single-celled, non-motile organelles found on the surface of most mammalian cells, and they serve as hubs for receiving and transducing extracellular signals, playing a critical role in the development and maintenance of neural homeostasis in the mammalian brain [81]. Primary cilia and astrocytes play important roles in regulating inflammation, and dysfunction of both is closely associated with the development of neuroinflammation and cognitive decline [82]. Exercise can improve the pathological condition of AD patients by promoting the formation of primary cilia and enhancing the neuroprotective function of astrocytes. Cao et al. demonstrated that exercise suppresses the MAPK cascade in rats with chronic cerebral hypoperfusion by increasing the number and length of primary cilia in astrocytes, leading to reduced JNK and ERK phosphorylation, a decrease in C3/GFAP-positive astrocytes and an increase in S100A10/GFAP-positive astrocytes. Consequently, activated astrocytes polarize from neurotoxic A1 toward neuroprotective A2, decreasing the release of inflammatory factors and increasing the release of neurotrophic factors, thereby exerting neuroprotective effects [83]. A preclinical study has shown that exercise can also upregulate histone H3K18 lactylation through the production of lactate, thereby promoting the polarization of microglia toward an anti-inflammatory phenotype to reduce neuroinflammation and improve cognitive function [84]. Furthermore, a cross-sectional community-based cohort study found that increased physical activity can reduce neuroinflammation by lowering plasma GFAP levels, thus slowing the progression of neurodegeneration—with particularly significant effects observed in non-APOE4 carriers [85].

As a key regulator of inflammation, miR-146a also mediates the improvement of CVD-AD comorbidity through exercise. In an animal study, aerobic exercise can upregulate miR-146a levels. miR-146a binds to the 3′ untranslated region (3′UTR) of TRAF6 mRNA and inhibits TRAF6 expression to downregulate toll-like receptor 4 (TLR4), while inhibiting the NF-κB signaling pathway to decrease the expression and secretion of inflammatory factors and chemokines, ultimately improving vascular inflammatory injury in AS mice and promoting angiogenesis [86]. Relevant studies indicate that exercise may cause a transient increase in miR-146a in HF patients, but the overall trend shows a significant decrease in miR-146a levels [87,88]. This may be because acute exercise itself constitutes a systemic inflammatory stimulus. During exercise, the body upregulates miR-146a levels to mitigate this exercise-induced inflammatory stress and drive angiogenesis. As the frequency of regular exercise increases, the body develops immune tolerance to this stimulus and the need for angiogenesis decreases, resulting in lower miR-146a levels and reduced inflammation. In AD patients, miR-146a is overexpressed in cerebrospinal fluid but underexpressed in plasma. Under these pathological conditions, miR-146a targets CFH and binds to its 3′UTR to downregulate CFH expression, thereby lifting its inhibition of the NF-κB pathway, worsening neuroinflammation in AD patients and creating a vicious cycle [63]. Previous studies have shown that acute resistance exercise can reduce circulating miR-146a levels [89]. Whether exercise alleviates neuroinflammation and improves cognitive dysfunction by targeting the miR-146a/CFH axis to increase CFH levels or restore miR-146a levels in the AD brain requires further mechanistic investigation.

**Figure 4:**
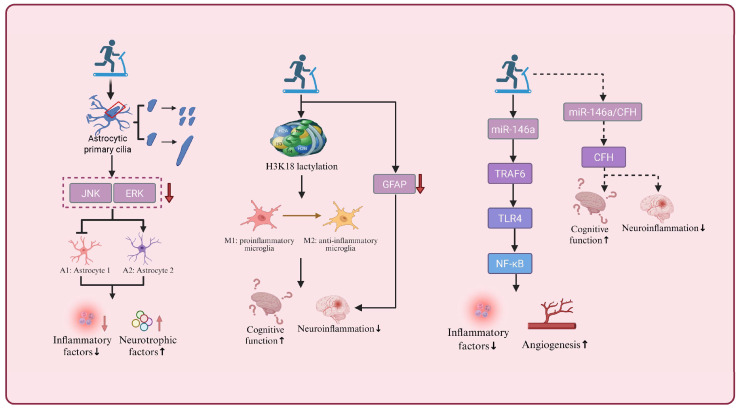
The mechanisms about exercise reduce inflammation in CVD-AD comorbidity. ⟶: This symbol represents a promoting effect. ⟞: This symbol represents an inhibitory effect. ↓: This symbol represents a horizontal decline. ↑: This symbol represents a horizontal rise or an enhancement of a function. Abbreviations: CFH, complement factor H; ERK, extracellular signal-regulated kinase; GFAP, glial fibrillary acidic protein; H3K18, histone H3 lysine 1; JNK, c-Jun N-terminal kinase; NF-κB, nuclear factor kappa-B; TLR4, toll-like receptor 4; TRAF6, TNF receptor-associated factor 6. Created in BioRender. Lu, Y. (2026) https://BioRender.com/bglunpw (accessed on 15 July 2026).

The mechanisms about exercise that reduce inflammation in CVD-AD comorbidity are summarized in Figure 4.

### 5.4. Exercise Protects Neuronal Structure and the BBB

At the microscopic molecular level, the degeneration of motor neurons in the brain and spinal cord caused by physical inactivity is associated with increased NfL levels [90]. NfL is a key indicator of neuronal axonal damage, loss, and changes in brain microstructure. High pNfL levels are common pathological features in patients with CVD and AD. Exercise exerts its neuroprotective effects by reducing pNfL levels [91,92]. HIIT has been shown to modulate pNfL by targeting the KYN pathway. The KYN pathway is the major catabolic pathway for tryptophan (TRP). This pathway can result in two outcomes: degradation into the neuroprotective kynurenic acid (KA) or into the neurotoxic quinolinic acid (QA). Exercise activates the KYN pathway and promotes its metabolic shift toward KA production, thereby shifting the QA/KA balance toward a neuroprotective state. Concurrently, it reduces pNfL levels, mitigating neuronal injury and improving comorbid pathological conditions in patients [93].

From the perspective of macro-level brain structure, exercise not only improves subcortical structures but also protects the cortex. A reduction in the dendritic complexity of neurons in the subcortical hippocampus and amygdala caused by tau protein pathology is an early sign of AD [94]. A preclinical study demonstrated that exercise can increase levels of Brain-Derived Neurotrophic Factor (BDNF) and Tropomyosin receptor kinase B (TrkB) in the hippocampus and amygdala of AD mice, activate the downstream AKT/ERK signaling pathway, and ultimately restore the dendritic architecture of the hippocampus and amygdala [95]. Furthermore, a clinical trial has shown that a one-year intervention involving aerobic exercise and stretching can improve cognitive function in sedentary healthy older adults by slowing the reduction in cortical thickness [96]. Similarly, a large-scale cross-sectional study indicated that exercise is associated with increased cortical thickness and brain volume, suggesting a potential neuroprotective effect [97].

BBB serves as a functional component of the neurovascular unit, playing a vital role in the prevention and treatment of CVD-AD comorbidity. Tight junction loosening, pericyte impairment, and low-density lipoprotein receptor-related protein 1 (LRP1) deficiency contribute to BBB dysfunction, which is a major factor in cognitive decline [98]. A recent animal study demonstrated that 16 weeks of treadmill training stimulates brain neurons to release miR-532-5p, which directly binds to the 3′UTR of the erythropoietin-producing hepatocellular carcinoma A4 (EPHA4) to suppress EphA4 expression, resulting in increased expression levels of pericyte-associated proteins PDGFRβ, NG2, and tight junction proteins. Concurrently, the exercise-regulated miR-532-5p/EPHA4 signaling pathway also enhances LRP1-mediated Aβ efflux clearance efficiency [99]. Therefore, exercise-induced miR-532-5p can repair the structure of the BBB and enhance its functional integrity. This mechanism can prevent harmful blood components such as fibrinogen and albumin from entering the brain tissue, thus improving the pathological state of the central nervous system at the level of physical defense.

**Figure 5:**
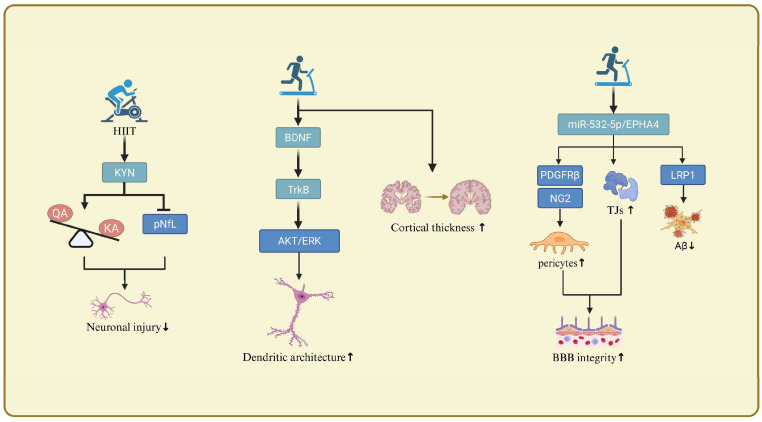
The mechanisms about exercise protect neuronal structure and the BBB in CVD-AD comorbidity. ⟶: This symbol represents a promoting effect. ⟞: This symbol represents an inhibitory effect. ↓: This symbol represents a horizontal decline. ↑: This symbol represents a horizontal rise or an enhancement of a function. Abbreviations: Aβ, amyloid-β; AKT, protein kinase B; BBB, blood–brain barrier; BDNF, brain-derived neurotrophic factor; EPHA4, erythropoietin-producing hepatocellular carcinoma A4; ERK, extracellular signal-regulated kinase; HIIT, high-intensity interval training; KA, kynurenic acid; KYN, kynurenine; LRP1, low-density lipoprotein receptor-related protein 1; NG2, neuron-glial antigen 2; PDGFRβ, platelet-derived growth factor receptor β; pNfL, plasma neurofilament light chain (plasma NfL); QA, quinolinic acid; TJs, tight junctions; TrkB, tropomyosin receptor kinase B. Created in BioRender. Lu, Y. (2026) https://BioRender.com/vah72i2 (accessed on 15 July 2026).

The mechanisms about exercise that protect neuronal structure and the BBB in CVD-AD comorbidity are summarized in Figure 5.

### 5.5. Exercise Improves Metabolic Disorders

As people age, metabolic dysfunction has gained wide attention in chronic diseases such as CVD, AD, and type 2 diabetes, with primary manifestations including IR, dyslipidemia, and obesity. Exercise offers multiple benefits in this setting. Numerous studies have shown that exercise can exert beneficial effects on peripheral insulin sensitivity. It enhances insulin signaling in skeletal muscle, activates AMP-activated protein kinase (AMPK) to promote glucose transport and metabolism, and increases mitochondrial biosynthesis and oxidative capacity, ultimately improving insulin sensitivity and reducing cardiovascular risk [100,101,102]. Meanwhile, exercise can also influence cognition by improving central nervous system IR. Evidence indicates that central nervous system IR is a key characteristic of AD patients, and insulin transport across the BBB is significantly reduced in this population [103]. Brown et al.’s voluntary running wheel exercise experiment in mice showed that acute exercise enhances insulin transport across the BBB, increases insulin vascular binding levels, and alters insulin receptor signaling in the central nervous system. These exercise effects exhibit sex differences: exercise significantly increased whole-brain insulin transport rates and insulin vascular binding levels in the olfactory bulb of male mice, whereas female mice showed no significant changes in whole-brain insulin transport rates and a significant decrease in insulin vascular binding levels in the hypothalamus. This may be related to factors including sex hormones and exercise intensity [104].

In addition, exercise improves metabolic function by regulating the secretion of exerkines, helping to slow the progression of CVD-AD comorbidity. Irisin is an exerkine mainly produced in skeletal muscle during exercise. It increases energy or adenosine triphosphate (ATP) production by promoting the conversion of white adipose tissue into brown adipose tissue, and activates the AMPK/mTOR signaling pathway to improve insulin sensitivity and reduce fat accumulation [105]. As exercise promotes irisin secretion, it also elevates levels of BDNF in the body. Increased BDNF concentrations can promote the generation of hippocampal neurons and synapses, thus enhancing cognitive function by improving their plasticity. In recent years, relevant cross-sectional studies have found that BDNF can also influence blood glucose homeostasis and insulin sensitivity, thereby improving metabolic health. This indicates that exercise-induced BDNF plays a multifaceted role in regulating metabolism and brain health [106]. In addition to these two exerkines, exercise also promotes the secretion of fibroblast growth factor 21 (FGF21). FGF21 is a hepatokine that regulates energy metabolism, maintains mitochondrial homeostasis, prevents CVD, and provides neuroprotection [107,108]. An animal study has shown that exercise-induced FGF21 not only acts directly on the cardiovascular system by reducing vascular endothelial injury and abnormal lipid accumulation, but also inhibits the NF-κB and AMPKα/AKT signaling pathways to lower lipid peroxidation products. By reducing oxidative stress and inflammation, it protects the BBB and optimizes the structure and function of cerebral blood vessels and neurons [109]. In summary, exercise plays a crucial role in synergistically improving CVD-AD comorbidity by enhancing insulin sensitivity, optimizing lipid metabolism, and promoting the secretion of exerkines, while simultaneously regulating the body’s energy and material metabolism.

**Figure 6:**
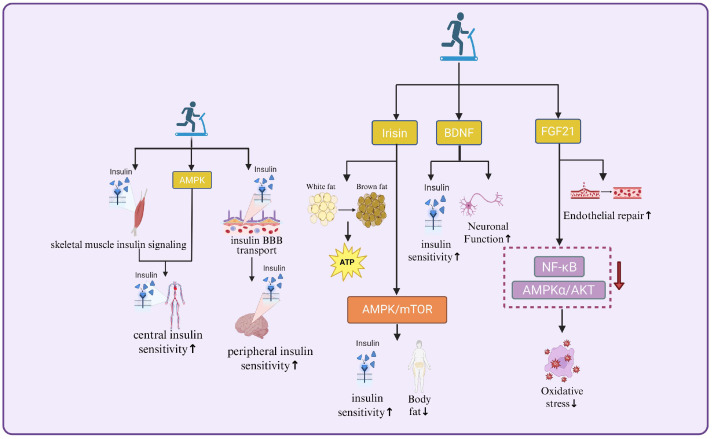
The mechanisms about exercise improve metabolic disorders in CVD-AD comorbidity. ⟶: This symbol represents a promoting effect. ↓: This symbol represents a horizontal decline. ↑: This symbol represents a horizontal rise or an enhancement of a function. Abbreviations: AKT, protein kinase B; AMPK, AMP-activated protein kinase; ATP, adenosine triphosphate; BBB, blood–brain barrier; BDNF, brain-derived neurotrophic factor; FGF21, fibroblast growth factor 21; mTOR, mammalian target of rapamycin; NF-κB, nuclear factor kappa-B. Created in BioRender. Lu, Y. (2026) https://BioRender.com/hy3d2d0 (accessed on 15 July 2026).

The mechanisms about exercise that improve metabolic disorder in CVD-AD comorbidity are summarized in Figure 6.

## 6. The Impact of Different Exercise Modalities on CVD and AD Comorbidity

The various molecular mechanisms mediated by exercise provide a theoretical basis for improving CVD-AD comorbidity. Extensive clinical evidence suggests that different exercise modalities may yield varying benefits for different diseases or different stages of the same disease. Current research on exercise modalities primarily focuses on aerobic exercise, resistance exercise, and combined exercise, as well as other forms such as Tai Chi, Baduanjin, and yoga Table A1. The Effects of Exercise on Blood Biomarkers and Molecular Signaling in CVD-AD Comorbidity.

The majority of these studies target a single disease (Table A2, Table A3 and Table A4). As an intermediate state between normal cognitive function and AD, mild cognitive impairment (MCI) has received significant attention in studies on exercise’s role in the early prevention of AD. Additionally, in the context of CVD, research mainly explores the effects of exercise on HF, AF, and hypertension. Based on these studies of individual diseases, we have inferred and summarized some appropriate specific exercise parameters. However, evidence-based recommendations specifically tailored for patients with CVD-AD comorbidity remain limited at present.

### 6.1. Aerobic Exercise

Aerobic exercise refers to regular, sustained, purposeful, and conscious physical activity performed under conditions of adequate oxygen supply. As a core clinical intervention, it offers distinct advantages in enhancing cardiac pump efficiency, improving vascular dilation, and boosting cardiopulmonary function. Based on multiple studies of older adults with cognitive impairment or AD, 6–12-month aerobic exercise interventions can significantly increase VO_2_peak, which serves as a measure of cardiorespiratory fitness and a key indicator for predicting prognosis in CVD patients [110,111,112]. Research indicates that exercise-induced improvements in cardiopulmonary endurance are positively related to the integrity of brain structures such as white matter [113]. Therefore, cardiopulmonary endurance may act as a link between the benefits of aerobic exercise and improvements in cognitive function. Moreover, aerobic exercise of moderate intensity has been shown to enhance cardiovascular function in middle-aged and older adults across all age groups, primarily through improvements in vasodilation, endothelial function, and microvascular function.

Most experimental data indicate that aerobic exercise is typically performed 3–5 times per week. For patients with CVD, it is recommended to maintain a frequency of at least 3 times per week, which is related to the maintenance of myocardial training stimulation and vasodilatory effects [114]. A single exercise session usually lasts 20–60 min. To maintain an active lifestyle, individuals should engage in at least 150 min of moderate-intensity aerobic exercise or 75 min of vigorous-intensity aerobic exercise each week. Clinically, continuous aerobic exercise modes involving larger muscle groups are mostly adopted, including brisk walking, cycling, treadmill walking, and swimming. Exercise intensity is a core indicator of exercise intervention. For patients with CVD and AD, an initial moderate intensity of 40–60% HRR is recommended; patients with better physical fitness can gradually transition to a high intensity of 80% HRR, under which exercise benefits may exceed those of lower-intensity exercise. It should be noted that CVD patients commonly take β-blockers, which can reduce resting and exercise heart rate, cardiac output, and myocardial contractility. Using traditional maximum heart rate (HR_max_) monitoring may result in exercise intensity higher than actually required, causing premature fatigue, hypotension, or even myocardial ischemia [115]. Studies have shown that β-blockers have no significant effect on the Rating of Perceived Exertion (RPE, Borg 6–20 Scale). Therefore, using RPE to control the intensity of patients’ aerobic exercise can better reflect their physiological stress, and it is recommended that RPE be set at 12–14.

### 6.2. Resistance Exercise

Resistance exercise is a form of physical activity that induces muscle contraction through external resistance; it also produces beneficial physiological and clinical effects on cardiovascular risk factors and CVD. In a study of patients with stage 1 hypertension, 9 weeks of resistance training reduced systolic and diastolic blood pressure by 4–8 mmHg, while also lowering total peripheral resistance and improving cardiovascular function [116]. Resistance exercise offers unique advantages in improving or maintaining muscle mass and strength. It may enhance cognitive function by improving brain structural plasticity in patients with cognitive impairment. One study showed that a 12-week resistance exercise intervention significantly increased the thickness of the parahippocampal cortex and the right entorhinal cortex. These two regions together form the core of memory and spatial cognition. By acting on these areas, resistance exercise effectively improves cognitive function in the hippocampal complex and slows neuronal loss in older adults at risk for MCI [117]. Similarly, Broadhouse et al. found that a 6-month progressive resistance exercise intervention protected AD-vulnerable hippocampal subregions, increased the structural plasticity of the hippocampus, and improved whole-body muscle strength [118].

To ensure the heart muscle has sufficient time to recover and to reduce the risk of acute cardiovascular events, patients with CVD and AD are recommended to engage in non-consecutive training 2–3 times per week, ideally with a day of rest in between sessions. The European Society of Cardiology recommends performing 1–3 sets per time, with 8–12 repetitions per set. For frail older adults, training may begin with 1 set per time, consisting of 10–15 repetitions, at a moderate intensity of 40–50% 1RM [119]. Once patients can perform 2 more repetitions of a given exercise during two consecutive resistance training sessions, the weight per set can be increased by 2–10% of 1RM, and later gradually increased to 70–80% of 1RM. Exercise styles may include dumbbells, push-ups, squats, machine weights, and resistance bands, forming a training program that targets 8–10 different exercises for major muscle groups. The most critical risk factor in resistance exercise for CVD patients is the Valsalva maneuver. This breathing pattern causes a sharp rise in intrathoracic pressure, which can trigger adverse events such as angina, arrhythmias, and microvascular rupture. Therefore, it is essential to maintain a rhythmic breathing pattern during exercise, exhaling during the exertion phase and inhaling during the relaxation phase [120]. Studies indicate that even short-term resistance exercise can yield health benefits for patients with CVD. For patients who find it difficult to perform aerobic exercise due to functional limitations or who dislike it, resistance exercise is a suitable exercise modality and may help improve exercise adherence [121,122].

### 6.3. Other Exercise

With advancing research, it has been found that traditional, single-modality aerobic or resistance exercise may not comprehensively improve patients’ functional abilities. Emerging exercise modalities such as HIIT, aerobic-resistance combined exercise, yoga, Tai Chi and Baduanjin have shown additional benefits. For CVD patients, combined exercise can improve cardiovascular function and enhance cardiorespiratory endurance [123]. For individuals with cognitive impairment, combined exercise not only improves executive function, memory, and overall cognitive function in older adults with MCI, but also enhances overall physical function and cardiovascular function in patients with AD. Furthermore, there is a dose–response effect between exercise benefits and intensity, meaning that higher-intensity exercise yields greater improvements than lower-intensity exercise [124,125,126,127]. Patients can adopt an exercise frequency of three times per week, with each session structured as a 10-min warm-up, 30 min of aerobic exercise, and 20 min of resistance training. The intensity of each component and the equipment used can be based on the guidelines for single aerobic and resistance exercise modes. It is again recommended to start at a moderate intensity. However, as combined aerobic-resistance exercise involves a variety of movement patterns, it may not be advantageous in terms of exercise adherence among older adults.

Traditional Chinese exercises such as Tai Chi and Baduanjin are characterized by low cost, ease of practice and high adherence, offering a new exercise intervention strategy for patients with the comorbidity of CVD and AD. Evidence indicates that Tai Chi can improve vascular dilation in older adults with CVD, and lower blood pressure and blood lipid levels, showing superior efficacy compared to traditional single-modality aerobic exercise. It also plays a role in improving cognitive function and memory during the early stages of AD prevention [128,129,130]. Baduanjin also plays a role in enhancing physical function and quality of life in older adults with HF [131]. Patients can practice Tai Chi or Baduanjin 3–5 times per week, for 45–60 min per session, at a moderate intensity of 40–60% HRR. Additionally, yoga is a form of physical exercise distinct from the above, primarily consisting of breath control, meditation, and relaxation exercises. It plays a significant role in reducing acute cardiovascular events, enhancing cognition, and alleviating anxiety and depression [132,133]. Yoga is characterized by low risk and high inclusivity, making it suitable for beginners or for use during relaxation phases. The recommended frequency is once a week, with each session lasting 30–60 min. Because Tai Chi, Baduanjin, and yoga all involve social interaction during practice, they offer an added benefit in improving patients’ mental health. It is worth highlighting that different intervention methods should be adopted for individuals at different stages of the disease. For patients with significant cognitive impairment, complex routines may lead to cognitive overload and increased anxiety. In such cases, simplifying the movements to reduce the learning intensity is necessary to achieve better outcomes.

## 7. Pharmacological Treatments and Nutritional Interventions for CVD and AD Comorbidity

### 7.1. Pharmacological Treatments

Cardiovascular dysfunction, chronic inflammation, and systemic metabolic disorders are key drivers of vascular pathology and neurodegenerative diseases. For the treatment of CVD and AD, clinical practice primarily relies on pharmacological strategies, which can be divided into three major categories: hemodynamic and vascular integrity modulators, neuroimmune and inflammatory cascade inhibitors, and metabolic remodeling and protein homeostasis protectors.

The main hemodynamic and vascular integrity modulators include the renin-angiotensin system (RAS) inhibitor candesartan, β-blockers, and the antiplatelet drug aspirin. Evidence indicates that candesartan can reduce Aβ levels in the brains of both hypertensive and non-hypertensive AD patients, enhance subcortical brain connectivity, and produce significant cognitive improvements [134]. β-blockers can improve cardiac function. For patients with heart failure with reduced ejection fraction (HFrEF), RAS inhibitors are appropriate for all age groups, whereas the effects of β-blockers vary depending on patient age. Specifically, in patients younger than 80 years of age, the use of β-blockers is associated with a reduction in all-cause mortality and cardiac mortality; however, this association was not observed in patients aged 80 years or older [135]. As an antiplatelet drug, aspirin’s primary value lies in preventing microembolism and maintaining cerebral blood flow. A large-scale clinical trial has shown that, in healthy older adults without underlying cardiovascular disease, daily low-dose aspirin does not reduce the incidence of Alzheimer’s disease or slow overall cognitive decline [136]. But for people with coronary heart disease (CHD) or those at very high cardiovascular risk, long-term (10 years or more) use of aspirin can significantly reduce the risk of AD [137].

The main neuroimmune and inflammatory cascade inhibitors are the anti-IL-1β antibody Canakinumab and colchicine. A large-scale randomized, placebo-controlled trial indicated that higher doses of Canakinumab, as an anti-inflammatory agent, can reduce IL-6 and CRP levels, as well as significantly lower the incidence of cardiovascular events [138]. However, due to its high cost and the potential risk of life-threatening sepsis, canatamab has not been adopted in cardiovascular treatment. Colchicine, another anti-inflammatory drug that is safer than canakinumab and less expensive, has been used to reduce the residual risk of cardiovascular events in the secondary prevention of coronary artery disease [139,140]. A clinical study has shown that, in patients with chronic coronary heart disease, low-dose colchicine can reduce the incidence of cardiovascular death, myocardial infarction, or stroke. It should be noted that the use of this medication may slightly increase the risk of pneumonia in patients [141].

Metabolic reprogramming and protein homeostasis protectors primarily include GLP-1 receptor agonists and sodium-glucose cotransporter-2 (SGLT2) inhibitors. GLP-1 receptor agonists were originally developed to treat diabetes and obesity. These include drugs such as liraglutide and semaglutide, which have been shown to cross the BBB and influence insulin signaling, and neuroinflammatory processes, along with amyloid and tau-related pathways, playing a significant role in the treatment of CVD and AD [142,143,144,145]. SGLT2 inhibitors also exhibit neuroprotective and metabolic protective effects. Evidence indicates that SGLT2 inhibitors induce mild ketosis by promoting fatty acid oxidation, increasing circulating levels of β-hydroxybutyrate, and serving as an alternative energy source to sustain mitochondrial ATP production in neurons during brain insulin resistance, thus mitigating Aβ-related oxidative damage [146]. In addition, compared with incretin mimetics, SGLT2 inhibitors significantly reduced the overall risk of dementia, and their efficacy was greater than that of GLP-1 receptor agonists [147].

### 7.2. Nutritional Interventions

Within the complex pathophysiological network of CVD and AD comorbidity, non-pharmacological nutritional interventions and natural products play a significant role in improving peripheral cardiovascular endothelial function and alleviating central nervous system neurodegenerative disorders.

The Mediterranean Diet (MDS), the DASH diet, and the MIND diet are common healthy dietary patterns that are highly recommended for their high content of polyunsaturated fatty acids and plant polyphenols [148]. A cross-sectional study revealed that the MDS is associated with AD biomarkers; it can reduce plasma NfL levels, and among APOE4 carriers, the negative correlation between the MDS and plasma p-tau181 is stronger than among non-carriers. The MDS also shows a negative correlation with plasma GFAP, but this correlation is observed only among participants aged 78 or older [149].

The nutritional supplements vitamins D and E play a role in maintaining the structural integrity of neurovascular units. As a steroid hormone, vitamin D can specifically bind to vitamin D receptors on the endothelial cells of cerebral microvessels. This transcriptional cascade directly upregulates and maintains the expression and stability of key tight junction proteins in the BBB [150]. On the other hand, vitamin E, as a classic fat-soluble antioxidant, can effectively slow the lipid peroxidation of LDL and HDL in the circulatory system, ultimately delaying the progression of peripheral atherosclerotic plaques [151].

Natural polyphenolic compounds extracted from plants are another powerful tool for improving cardiovascular and cerebrovascular inflammation and addressing impaired amyloid clearance. Resveratrol is a potent SIRT1 agonist. The activated SIRT1 signaling pathway not only protects the cardiovascular system by increasing the activity of eNOS, but also upregulates the transcriptional expression of ADAM10 in central neurons, promoting the cleavage of APP into non-amyloid pathways and reducing Aβ production [152]. Meanwhile, curcumin is able to cross the BBB and bind directly to small-molecule Aβ, inhibiting its aggregation and fibril formation both in vitro and in vivo [153]. Furthermore, at the molecular level, curcumin can inhibit the NF-κB and NLRP3 pro-inflammatory pathways while activating the Nrf2 antioxidant pathway, ultimately reducing the release of inflammatory cytokines by microglia [154].

### 7.3. Coordination of Exercise with Pharmacy and Nutritional Interventions

In the treatment of CVD and AD comorbidity, multimodal interventions are becoming the leading trend. The combined use of exercise with medication, dietary interventions, or natural compounds produces complex effects within the body, manifesting as synergistic effects, parallel benefits, or attenuating effects. For example, a Finnish study on the prevention of cognitive impairment and disability in older adults found that the group receiving a combined intervention of aerobic and resistance exercises, MDS, and cardiovascular risk management showed significantly greater improvements in overall cognitive function, executive function, and processing speed compared to those receiving a single intervention, demonstrating a strong synergistic effect [155]. Similarly, research indicates that combining GLP-1 agonists with exercise provides additive and complementary benefits, promoting sustainable fat loss while mitigating adverse metabolic adaptations [156]. In another randomized clinical trial, aerobic and resistance exercise significantly improved cognitive function in older adults with mild cognitive impairment; yet, the addition of high-dose vitamin D did not further enhance the cognitive benefits of exercise, indicating a parallel rather than additive effect [124]. Moreover, the combination of exercise and nutritional interventions can also produce a blunting effect. The study found that supplemental resveratrol actually offset the cardiovascular benefits (including improved cardiorespiratory endurance and blood pressure-lowering effects) resulting from exercise training in older men [157].

## 8. Conclusions and Future Perspectives

The comorbidity of CVD and AD involves cross-organ pathological transmission via the heart-brain axis. As the core of the peripheral circulatory system and an endocrine organ, cardiac dysfunction not only leads to chronic cerebral hypoperfusion but also accelerates Aβ aggregation, Tau phosphorylation, and axonal damage through oxidative stress and chronic inflammation. Metabolic imbalance (mainly IR and lipid disorders) and genetic risk factors (exemplified by APOE4) also play a critical role in the development of this comorbidity. According to existing research evidence, blood biomarkers associated with CVD-AD comorbidity perform different roles at different stages of the comorbidity. CVD pathology generally precedes AD. The blood biomarkers p-tau217 and NT-proBNP can be used to identify cognitive decline in CVD patients at an early stage and to predict an individual’s future risk of AD. NfL serves to assess brain damage in the later stages of comorbidity and can also be used to evaluate the effectiveness of interventions. GFAP is mainly related to the inflammatory state in the early stages of comorbidity and can capture the transition from vascular inflammation to neuroinflammation. miRNAs serve as signaling molecules with relatively high stability; their levels reflect the body’s metabolic state in the context of comorbidity and have roles in the diagnosis and prognosis of CVD-AD comorbidity.

Exercise can improve the comorbidities of CVD and AD. It acts on blood biomarkers and other factors by regulating multiple signaling pathways, leading to various downstream biological effects. Different types of exercise have different effects on comorbidity. Aerobic exercise is the most common and widely used form in clinical practice, and it effectively improves cardiovascular and cardiopulmonary function. Resistance training offers additional benefits, including enhanced muscle strength and repair of brain structure. Traditional single-modality exercise interventions may have limitations. Consequently, growing research is adopting combined aerobic and resistance exercise interventions to maximize the benefits. However, patient adherence to this approach remains a concern. With increasing cultural exchange, yoga and traditional Chinese activities such as Tai Chi and Baduanjin are being integrated into exercise intervention strategies, providing additional benefits to psychological well-being and quality of life.

Although research on exercise interventions is deepening, most studies have focused on single diseases, and evidence regarding the effects of exercise on comorbidities remains lacking. In clinical practice for CVD and AD, exercise interventions rarely occur in isolation but are typically combined with standard pharmacological treatments such as aspirin, β-blockers, angiotensin-converting enzyme inhibitors, or nutritional interventions like the MED, vitamin D supplements, and natural compound supplements. Against this backdrop, although there are potential interactions between exercise interventions and pharmacological or nutritional factors, the current clinical evidence regarding CVD and AD comorbidity remains limited. Therefore, the available data are insufficient to support clinically actionable conclusions regarding multimodal synergistic interventions or precision-tailored exercise prescriptions. Future exploratory clinical trials are needed to assess the actual impact of different exercise interventions on comorbidity and to gradually investigate potential interactions with pharmacological or nutritional interventions. In the long term, with the advancement of artificial intelligence and multi-omics technologies, the identification of more specific biomarkers for comorbidity will provide fundamental scientific insights for steadily advancing and exploring personalized intervention strategies for patients with CVD-AD comorbidity, with the ultimate goal of improving patients’ quality of life.

## Data Availability

No new data were created or analyzed in this study. Data sharing is not applicable to this article.

## Abbreviations

3′UTR	3′ untranslated region
AD	Alzheimer’s disease
AEP	asparagine endopeptidase
AF	atrial fibrillation
AMPK	AMP-activated protein kinase
APOE	apolipoprotein E
APP	amyloid precursor protein
AQP4	aquaporin channel 4
AS	atherosclerosis
ATP	adenosine triphosphate
Aβ	amyloid-β
BACE1	β-secretase
BBB	blood–brain barrier
BDNF	brain-derived neurotrophic factor
BNP	brain natriuretic peptide
CAD	coronary artery disease
CHIP	carboxyl terminus of HSC70-interacting protein
CHOP	CCAAT/enhancer-binding protein homologous protein
CHD	coronary heart disease
CVD	cardiovascular disease
CSF	cerebrospinal fluid
EPHA4	erythropoietin-producing hepatocellular carcinoma A4
ER	endoplasmic reticulum
ET-1	endothelin-1
EVs	extracellular vesicles
FGF21	fibroblast growth factor 21
GFAP	Glial fibrillary acidic protein
GSK-3β	glycogen synthase kinase-3β
HDL	high-density lipoprotein
HF	heart failure
HIF-1α	hypoxia-inducible factor-1α
HIIT	high-intensity interval training
HIPK1	Homeodomain-interacting protein kinase 1
HRmax	heart rate (maximum heart rate)
HRR	heart rate reserve
HSP70	heat shock protein 70
HFrEF	heart failure with reduced ejection fraction
IDE	insulin-degrading enzyme
IL-1β	interleukin-1β
IL-6	interleukin-6
IR	insulin resistance
KA	kynurenic acid
LRP1	low-density lipoprotein receptor-related protein 1
MCI	mild cognitive impairment
MESA	Multiethnic Study of Atherosclerosis
MDS	Mediterranean Diet
miRNAs	MicroRNAs
MoCA	Montreal Cognitive Assessment
mtDNA	mitochondrial DNA
NfL	Neurofilament light chain
NFT	neurofibrillary tangles
NO	nitric oxide
NT-proBNP	N-terminal pro-B-type natriuretic peptide
p-tau	phosphorylated tau
PIK3R2	phosphoinositide-3-kinase regulatory subunit 2
pNfL	plasma NfL
QA	quinolinic acid
RAS	renin-angiotensin system
RM	repetition maximum
ROS	reactive oxygen species
RPE	rating of perceived exertion
SPRED1	sprouty related evh1 domain containing 1
SGLT2	sodium-glucose cotransporter-2
TLR4	toll-like receptor 4
TNF-α	tumor necrosis factor-α
TRP	tryptophan
TrkB	Tropomyosin receptor kinase B
UPR	unfolded protein response
VEGF	vascular endothelial growth factor
WMH	white matter hyperintensity

## Appendix A

**Table A1:** The Effects of Exercise on Blood Biomarkers and Molecular Signaling in CVD-AD Comorbidity.

Exercise Functions	Blood Biomarkers	Research Model	Level of Evidence	Molecular Signaling	References
Exercise Repairs Cerebral Hypoperfusion and Endothelial Injury	NT-proBNP	Male SD Rats Model	Preclinical Research	NPR-A, NPR-C, BNP/cGMP	[67]
		HF Patients Model	Clinical Trial	BNP, NT-proBNP	[68]
	miR-126	Male SD Rats Model	Preclinical Research	SPRED1, PIK3R2, PI3K/AKT/eNOS, MAKP, VEGF	[73,74]
	miR-222	exercised mice models	Preclinical Research	HMBOX1, P27, HIPK1	[75,76]
Exercise Accelerates Aβ Clearance and Inhibits Tau Hyperphosphorylation	Tau, Aβ	male SD rats and AD mice model	Preclinical Research	AQP4, Tau, Aβ	[2]
		AD mice model	Preclinical Research	PI3K/Akt, UCHL-1, APP, BACE1, HSC70, CHIP	[80]
Exercise Reduces Inflammation	GFAP	CCH rats model	Preclinical Research	JNK, ERK	[83]
		Middle-Aged and Older Adults with Good Cognitive Health	Observational Human Study	GFAP in plasma	[85]
	-	AD mice model	Preclinical Research	H3K18la	[84]
	miR-146a	AS mice model	Preclinical Research	miR-146a/TRAF6/TLR4/NF-κB	[86]
		male HFrEF patients	Clinical Trial	miR-146a	[88]
Exercise Protects Neuronal Structure and The BBB	NfL	MS Patients Model	Clinical Trial	KYN, pNfL	[93]
	-	AS mice model	Preclinical Research	BDNF, TrkB, AKT/ERK	[95]
		Sedentary Health in Older Adults	Clinical Trial	-	[96]
		Healthy Middle-Aged and Older Adults	Observational Human Study	-	[97]
	miR-532-5p	AS mice model	Preclinical Research	miR-532-5p/EPHA4, PDGFRβ, NG2, LRP1	[99]
Exercise Improves Metabolic Disorder	-	young, male and female CD-1 mice	Preclinical Research	insulin	[104]
		pancreatic cancer cells lines	Preclinical Research	Irisin, AMPK/mTOR	[105]
		Healthy Adults	Observational Human Study	BDNF	[106]
		diabetic and aging mice	Preclinical Research	FGF21, NF-κB, AMPKα-AKT	[109]

Abbreviations: AKT, protein kinase B; AMPK, AMP-activated protein kinase; APP, amyloid precursor protein; AQP4, aquaporin channel 4; Aβ, amyloid-β; BACE1, β-secretase; BDNF, brain-derived neurotrophic factor; BNP, brain natriuretic peptide; cGMP, cyclic guanosine monophosphate; CHIP, carboxyl terminus of HSC70-interacting protein; eNOS, endothelial nitric oxide synthase; EPHA4: erythropoietin-producing hepatocellular carcinoma A4; ERK, extracellular signal-regulated kinase; FGF21, fibroblast growth factor 21; GFAP, Glial fibrillary acidic protein; H3K18la, Histone H3 Lysine 18 lactylation; HIPK1, homeodomain-interacting protein kinase 1; HMBOX1, homeobox containing 1; HSP70, heat shock protein 70; JNK, c-Jun n-terminal kinase; TrkB, Tropomyosin receptor kinase B; KYN, Kynurenine; LRP1, low-density lipoprotein receptor-related protein 1; mTOR, mammalian target of rapamycin; MAPK, Mitogen-Activated Protein Kinase; NF-κB, nuclear factor kappa-B; NG2, neuron-glial antigen 2; NPR-A, natriuretic peptide receptor A; NPR-C, natriuretic peptide receptor C; NT-proBNP, N-terminal pro-B-type natriuretic peptide; p27, kininase inhibitory protein 1; PDGFRβ, platelet-derived growth factor receptor β; PI3K, phosphoinositide 3-kinase; PIK3R2, phosphoinositide-3-kinase regulatory subunit 2; pNfL, plasma NfL; SPRED1, sprouty related evh1 domain containing 1; TLR4, toll-like receptor 4; TRAF6, TNF receptor-associated factor 6; UCHL-1, ubiquitin C-terminal hydrolase L1; VEGF, vascular endothelial growth factor.

**Table A2:** Research on the effects of aerobic exercise on cardiovascular disease and Alzheimer’s disease.

Exercise Types	Disease Types	Model	Intervention	Measures	Results	References
climbing stairs, jogging, brisk walking, and cycling, Tai Chi	preHTN	Average age of 49.3 Middle-aged and older adults with preHTN	60 min per time, 4 times per week, moderate intensity, 12 months	12-month SBP, 24-h SBP, nighttime SBP, nighttime HR	12-month SBP↓, 24-h SBP↓, nighttime SBP↓, nighttime HR↓ (Tai Chi > Aerobic exercise)	[128]
Aerobic exercise, Tai Chi’s Bafa Wubu	CAD	Average age of 62 Older adults with CAD	60–75 min per time, 3 times per week, moderate intensity (40–60% HRR), 12 weeks	Endothelial function: NO, ET, VCAM-1, ICAM-1 Lipid profile: HDL, TC, TG, LDL	NO↑ (indicating vasodilation; no significant difference between the two groups), ET↓ (an indicator of vasoconstriction; no significant difference between the two groups), VCAM-1 and ICAM-1↓ (indicating inflammation and endothelial function; Tai Chi showed a trend toward better results than aerobic exercise), HDL↑ (significantly increased in aerobic exercise; no significant change in Tai Chi), TC: no significant change, TG: no significant change, LDL: no significant change	[129]
Swimming	CVD	Age: >55 Older adults with normal BP	45 min per time, 2–3 times per week, intensity based on individual ability, 8 weeks	%FMD, CVC, QOL	%FMD↑ (reflects large vessel and endothelial function; no significant change), CVC↑ (reflects microvascular function; no significant change), QOL↑	[158]
Walking, stationary biking + DASH diet	CIND, CVD	Average age of 65.4 Older adults at risk of CIND and CVD	35 min per time, 3 times per week, moderate intensity, 6 months	Executive function, memory/verbal fluency, CDR-SB, 6MWT distance, CVD risk	Executive function↑ (remained better to the non-exercise group at the 1-year follow-up); memory/verbal fluency: no significant; CDR-SB↓; 6MWT distance↑ (with a slight decline at the 1-year follow-up); CVD risk↓	[159]
Rehabilitation treadmill	MCI	Age: 34–79 Patients with MCI following a stroke	40 min per time, 5 times per week, moderate intensity (65% HRmax), 2 weeks	n-back task results, MoCA scores, visuospatial/executive function, delayed recall	3-back accuracy↑, MoCA total score↑, visuospatial/executive function↑, delayed recall↑	[160]
Brisk walking	aMCI	Age: 55–80 Older adults with MCI	25–40 min per time, 3–5 times per week, moderate intensity (75–85%HRmax) to high intensity (85–90%HRmax), 1 year	CVMR, VO_2peak_, memory function, executive function	Hypocapnia: CVMR↑, Hypercapnia: CVMR↓, VO_2peak_↑, Memory/executive function↑ (slight improvement)	[110]
brisk walking, cycling, etc.	AD	Average age of 64.9 Older adults at risk of AD	3 times per week, ≥150 min per week, moderate intensity, 26 weeks	CTSB, BDNF, klotho, PUFAs, sphingolipids	CTSB↑ (positively correlated with cognitive function), sphingolipids↓, BDNF↓ (correlated with changes in sphingolipids), klotho: no significant change (positively correlated with cardiopulmonary function), PUFAs↑	[161]
Aerobic walking, group dance	AD	Age: 60–79 Healthy older adults	60 min per time, 3 times per week, 60–75%HRmax, 6 months	White matter microstructure, cognitive function, cardiopulmonary function (VO_2peak_)	white matter plasticity↑, cognitive function↑, cardiorespiratory fitness↑ (aerobic walking > group dance)	[111]
Treadmill walking	AD	Average age of 72.9 Older adults with normal cognitive function but elevated or subthreshold levels of brain Aβ	≤50 min per time, 3–5 times per week, 60 min per week (increase by 21 min per week until reaching 150 min per week), 40–55% HRR (increase by 10% HRR every 3 months), 52 weeks	Brain Aβ burden, total brain volume, hippocampal volume, cognitive performance, VO_2peak_	Brain Aβ burden, total brain volume, hippocampal volume and cognitive performance: no significant changes, VO_2peak_↑	[112]

↓: This symbol represents a horizontal decline. ↑: This symbol represents a horizontal rise or an enhancement of a function. Abbreviations: 6MWT, Six-Minute Walk Test; AD, Alzheimer’s disease; aMCI, amnestic mild cognitive impairmen; BDNF, brain-derived neurotrophic factor; BP, blood pressure; CAD, coronary artery disease; CDR-SB, Clinical Dementia Rating-Sum of Boxes; CIND, cognitive impairments with no dementia; CTSB, cathepsin B; CVC, cutaneous vascular conductance; CVD, cardiovascular disease; CVMR, cerebral vasomotor reactivity; ET, endothelin; FMD: flow-mediated dilation; HDL, high-density lipoprotein; HR, heart rate; HRmax, maximum heart rate, HRR, heart rate reserve; ICAM-1, intercellular adhesion molecule-1; LDL, low-density lipoprotein; MCI, mild cognitive impairment; MoCA, Montreal Cognitive Assessment; NO, nitric oxide; preHTN, prehypertension; PUFAs, polyunsaturated free fatty acids; QOL, quality of life; SBP, systolic blood pressure; TC, total cholesterol; TG, triglycerides; VCAM-1, vascular cell adhesion molecule-1; VO_2peak_, peak oxygen uptake.

**Table A3:** Research on the effects of Resistance exercise on cardiovascular disease and Alzheimer’s disease.

Exercise Types	Disease Types	Model	Intervention	Measures	Results	References
Resistance exercise (bench press, hack squat, latissimus dorsi pulldown, leg extension, seated row, leg curl, and plank resistance exercises)	E/S1H	Average age of 54 Older adults with E/S1H	40 min per time, 3 times per week, 10–12 RM load, 9 weeks	SBP, cSBP, DBP, cDBP, FMD, cfPWV, BRS, HRV, CO, TPR, CRP	SBP↓, cSBP↓, DBP↓, cDBP↓, FMD↑ (reflecting vascular endothelial function), cfPWV: no significant change, BRS: no significant change, HRV: no significant change, CO↑, TPR↓, CRP: no significant change	[116]
Dumbbell and resistance band training	AMI	Average age of 55 Older adults with AMI	10–12 reps per set, 4 sets per time, 4 times per week, 60% MVC, 4 weeks	FMD of the brachial artery, plasma vWF, VO_2peak_	FMD of the brachial artery↑, plasma vWF↓ (reflecting the extent of endothelial damage), VO_2peak_↓	[121]
Progressive resistance training	HF	Average age of 44.11 Middle-aged adults with end-stage HF	6–10 reps per set, 6 sets per time, 3 times per week, 60–80% MVC, RPE ≤ 13, 4 weeks	FMD of the brachial artery	FMD of the brachial artery↑	[122]
Resistance training (Bench press, leg press, wide-grip lat pulldown, abduction machine, bicep curls, adduction machine, shoulder press, and heel raises) Aerobic (training Treadmill)	CHF	Average age of 55 Middle-aged and older adults with CHF and LVEF ≤ 45%	8–10 reps per set, 2 sets per time, 3 times per week, Borg RPE: 3–5; first 8 weeks: resistance training, next 16 weeks: combination of resistance and aerobic training; 24 weeks total 30 min/session, 3 times/week, 65–85% HRmax, Borg RPE: 3–5; first 8 weeks: aerobic training; next 16 weeks: combined resistance and aerobic training; 24 weeks	VO_2peak_, upper-body 1RM, QOL, SBP_peak_, DBP_peak_	VO_2peak_↑ (significant increase in the resistance training group; no significant change in the aerobic training group), upper-body 1RM↑ (significant increase in both groups; resistance training > aerobic training), QOL: no significant change, SBP_peak_ and DBP_peak_ ↓ (significant decrease only in the resistance training group)	[162]
progressive lower-body resistance training (Leg presses, leg curls, leg extensions, calf raises, etc.)	MCI	Age:60–85 Older adults at risk of MCI	45 min per time, 2–3 times per week, 70–85% of 1RM, 12 weeks	Reaction time, total cortical thickness, right entorhinal cortex thickness	Reaction time↓ (significantly in the high-risk MCI group), total cortical thickness: no significant change, right entorhinal cortex thickness↑ (significantly in the high-risk MCI group)	[117]
progressive Resistance training (Bench press, leg press, seated row, standing hip abduction, knee extension)	MCI	Average age of 69.5 Older adults with MCI	8 reps per set, 5–6 sets per time, 2–3 times per week, 80% of 1RM, 6 months	Executive function, hippocampal plasticity, muscle strength	executive function↑, hippocampal plasticity↑, neuroprotection in AD-vulnerable regions↑, connections between the putamen and hippocampus↑, overall strength↑	[118]

↓: This symbol represents a horizontal decline. ↑: This symbol represents a horizontal rise or an enhancement of a function. Abbreviations: AD, Alzheimer’s disease; AMI, acute myocardial infarction; BRS, cardiovagal baroreflex sensitivity; cDBP, central diastolic blood pressure; cfPWV, carotid-femoral pulse wave velocity; CHF, chronic heart failure; CO, cardiac output; CRP, C-reactive protein; cSBP, central systolic blood pressure; DBP, diastolic blood pressure; E/S1H, stage-1 hypertension; FMD, flow-mediated dilation; HF, heart failure; HRmax, maximum heart rate; HRV, heart rate variability; LVEF, left ventricular ejection fraction; MCI, mild cognitive impairment; MVC, maximum voluntary contraction; QOL, quality of life; RM, repetition maximum; RPE, rating of perceived exertion; SBP, systolic blood pressure; TPR, total peripheral resistance; VO_2peak_, peak oxygen uptake; vWF, von Willebrand factor.

**Table A4:** Research on the effects of Other exercise on cardiovascular disease and Alzheimer’s disease.

Exercise Types	Disease Types	Model	Intervention	Measures	Results	References
HIIT	AF	Average age of 69 Older adults with AF	2 training blocks of 8 min each (30 s of high-intensity exercise + 30 s of recovery), 80–100% of maximum power output, 2 times per week, 12 weeks	6MWT distance, QOL, resting HR, time of AF, PA level	6MWT distance↑, QOL↑, PA level↑, resting HR↓, time of AF↓	[163]
Aerobic training + Resistance training Stationary Bike	HF	Average age of 70 Older adults with HF	30–60 min per time, 3 times per week, 50–70% $\dot{V}$O_2max_, 12 months Seven types of weight training equipment 12–15 reps per set, 1–2 sets per day, 3 times per week, 60% of 1RM, 12 months	Modified PacKer score, VO_2peak_, NYHA class, E/e′, GSA	modified PacKer score: no significant change, VO_2peak_↑, NYHA class↓ (reflecting cardiac function), E/e′: no significant change (reflecting diastolic function), GSA: no significant (reflecting quality of life).	[123]
Aerobic training + Resistance training + Vitamin D supplements	MCI	Average age of 73.1 Older adults with MCI	Aerobic training + Resistance training + Vitamin D supplements 90 min per time, 3 times per week, 20 weeks Vitamin D supplementation: 10,000 IU per time, 3 times per week	ADAS-Cog-13 score, executive function	ADAS-Cog-13 score↓, executive function↑, vitamin D showed no significant benefit for the exercise program	[124]
Aerobic training + Resistance training Tai Chi	MCI	Age: ≥50 Older adults with MCI	60 min per time, 3 times per week, intensity: 4.3–5.5 METs (RPE: 6–20), 24 weeks Tai Chi 60 min per session, 3 times per week, intensity: 3.24 METs (RPE: 6–20), 24 weeks	MoCA-HK scores, executive function, memory, physical function, mental health	MoCA-HK score↑ (associated with overall cognitive function; Tai Chi > combined exercise), executive function↑ (Tai Chi > combined exercise), memory↑, physical function↑, mental health↑	[125]
Aerobic training (Stationary bike, treadmill, arm-crank on specific ergometer) + Resistance training (Bench press, leg curls, leg extensions, wide-grip lat pulldown, leg press)	AD	Average age of 79 Older adults with AD	45 min/time, 3 times/week; the first week: 65%HRmax, increasing by 5% every 4 weeks; 6 months 30 min/time, 12 reps/set, 85% of 1RM, 5% increase in load, 6 months	%FMD, BF, VEGF, 6MWT distance, PPT score	%FMD↑ (reflecting endothelial function), BF↑, VEGF↑, 6MWT distance↑, PPT score↑	[126]
Aerobic training (Walking) + Resistance training (Lower-body strength training)	AD	Average age of 82.3 Older adults with AD	30 min/time, 3 times/week, 12 weeks at low intensity (57–63% HRmax, RPE: 9–11), 12 weeks at high intensity (71–89% HRmax, RPE: 13–16), 24 weeks 30 min/time, 3 times/week, 12 weeks at low intensity (RPE: 9–11), 12 weeks at high intensity (RPE: 13–16), 24 weeks	6MWT distance, walking speed, lower-body muscle strength, cognitive function	6MWT distance↑, walking speed↑ (high-intensity phase > low-intensity phase), lower-body muscle strength and cognitive function: no significant changes	[127]
Yoga (Includes breathing exercises, meditation, and relaxation techniques)	AMI	Average age of 53.4 Older adults with AMI	75 min per time, 13 sessions, 12 weeks	Incidence of MACE, QOL	Incidence of MACE: no significant change (there was a decrease trend), QOL↑	[132]
Yoga	AD	Average age of 62.8 Older women at risk of AD and CVD	60 min per time, once a week, 12 weeks	GMV, anxiety, depression, resilience, memory	GMV↑, anxiety↓, depression↓, resilience: no significant change, memory: no significant change	[133]
Baduanjin	HF	Average age of 60.5 Older adults with HF	45 min per time, moderate intensity, 12 weeks	6MWT distance, QOL, cardiopulmonary function	6MWT distance↑, QOL↑, cardiopulmonary function: no significant change	[131]
Tai Chi	MCI	Age: ≥60 Older adults with MCI	45–50 min per time, 45–75% HRR, 12 weeks	MoCA scores, MMSE scores, executive function, memory	MoCA score↑, MMSE score↑, executive function↑, memory↑	[130]

↓: This symbol represents a horizontal decline. ↑: This symbol represents a horizontal rise or an enhancement of a function. Abbreviations: 6MWT, Six-Minute Walk Test; AD, Alzheimer’s disease; ADAS-Cog-13, Alzheimer’s Disease Assessment Scale-Cognitive Subscale (13 items; AF, atrial fibrillation; AMI, acute myocardial infarction; E/e′, ratio of transmitral Doppler early filling velocity to tissue Doppler early diastolic mitral annular velocity (diastolic function); FMD, flow-mediated dilation; GMV, brain gray matter volume; GSA, global self-assessment; HF, heart failure; HIIT, high-intensity interval training; HRmax, maximum heart rate; HRR, heart rate reserve; MACE, major adverse cardiovascular events; MCI, mild cognitive impairment; MET, metabolic equivalent of task; MMSE, Mini-Mental State Examination; MoCA, Montreal Cognitive Assessment; MoCA-HK, Montreal Cognitive Assessment-Hong Kong version; NYHA, New York Heart Association; PA, physical activity; PPT, physical-performance test; QOL, quality of life; RM, repetition maximum; RPE, rating of perceived exertion; VEGF, vascular endothelial growth factor; $\dot{V}$O_2max_, peak oxygen consumption (maximum oxygen uptake); VO_2peak_, peak oxygen uptake.

## References

[1] Scheltens P., Blennow K., Breteler M.M., de Strooper B., Frisoni G.B., Salloway S., Van der Flier W.M. (2016). Alzheimer’s disease. Lancet.

[2] Feng S., Wu C., Zou P., Deng Q., Chen Z., Li M., Zhu L., Li F., Liu T.C.-Y., Duan R. (2023). High-intensity interval training ameliorates alzheimer’s disease-like pathology by regulating astrocyte phenotype-associated AQP4 polarization. Theranostics.

[3] Newman A.B., Fitzpatrick A.L., Lopez O., Jackson S., Lyketsos C., Jagust W., Ives D., Dekosky S.T., Kuller L.H. (2005). Dementia and Alzheimer’s disease incidence in relationship to cardiovascular disease in the Cardiovascular Health Study cohort. J. Am. Geriatr. Soc..

[4] Saeed A., Lopez O., Cohen A., Reis S.E. (2023). Cardiovascular Disease and Alzheimer’s Disease: The Heart-Brain Axis. J. Am. Heart Assoc..

[5] Silvani A., Calandra-Buonaura G., Dampney R.A., Cortelli P. (2016). Brain-heart interactions: Physiology and clinical implications. Philos. Trans. A Math. Phys. Eng. Sci..

[6] Livingston G., Huntley J., Liu K.Y., Costafreda S.G., Selbæk G., Alladi S., Ames D., Banerjee S., Burns A., Brayne C. (2024). Dementia prevention, intervention, and care: 2024 report of the Lancet standing Commission. Lancet.

[7] Zhao Y., Gong C.X. (2015). From chronic cerebral hypoperfusion to Alzheimer-like brain pathology and neurodegeneration. Cell. Mol. Neurobiol..

[8] Toyli A., Shaik A., Zhao C., Chen Q.-H., Sha Q., Zhou W. (2025). The heart-brain axis: Unraveling the interconnections between cardiovascular and alzheimer’s diseases. Front. Cardiovasc. Med..

[9] Ungvari Z., Toth P., Tarantini S., Prodan C.I., Sorond F., Merkely B., Csiszar A. (2021). Hypertension-induced cognitive impairment: From pathophysiology to public health. Nat. Rev. Nephrol..

[10] Bolaji O., Bahar Y., Lohana S., Bahar A.R., Lawrence I., Mazimba S. (2025). The heart-brain axis: Neurocognitive frailty in heart failure. J. Neurol..

[11] Nakase T., Tatewaki Y., Thyreau B., Odagiri H., Tomita N., Yamamoto S., Takano Y., Muranaka M., Taki Y. (2023). Impact of atrial fibrillation on the cognitive decline in Alzheimer’s disease. Alzheimers Res. Ther..

[12] Marasinghe C.K., Youn K., Ho C.-T., Jun M. (2026). Targeting the cardiovascular-alzheimer’s disease axis: The promise of marine bioactive peptides. Mar. Drugs.

[13] Maroofi A., Moro T., Agrimi J., Safari F. (2022). Cognitive decline in heart failure: Biomolecular mechanisms and benefits of exercise. Biochim. Biophys. Acta Mol. Basis Dis..

[14] Alexander C., Li T., Hattori Y., Chiu D., Frost G.R., Jonas L., Liu C., Anderson C.J., Wong E., Park L. (2022). Hypoxia Inducible Factor-1α binds and activates γ-secretase for Aβ production under hypoxia and cerebral hypoperfusion. Mol. Psychiatry.

[15] Barcena M.L., Aslam M., Pozdniakova S., Norman K., Ladilov Y. (2022). Cardiovascular inflammaging: Mechanisms and translational aspects. Cells.

[16] Perrone L., Valente M. (2021). The Emerging Role of Metabolism in Brain-Heart Axis: New Challenge for the Therapy and Prevention of Alzheimer Disease. May Thioredoxin Interacting Protein (TXNIP) Play a Role?. Biomolecules.

[17] San-Millan I. (2023). The Key Role of Mitochondrial Function in Health and Disease. Antioxidants.

[18] Parodi-Rullan R.M., Javadov S., Fossati S. (2021). Dissecting the crosstalk between endothelial mitochondrial damage, vascular inflammation, and neurodegeneration in cerebral amyloid angiopathy and alzheimer’s disease. Cells.

[19] Waigi E.W., Webb R.C., Moss M.A., Uline M.J., McCarthy C.G., Wenceslau C.F. (2023). Soluble and insoluble protein aggregates, endoplasmic reticulum stress, and vascular dysfunction in alzheimer’s disease and cardiovascular diseases. Geroscience.

[20] Cyr B., Cabrera Ranaldi E.D.L.R.M., Hadad R., Dietrich W.D., Keane R.W., de Rivero Vaccari J.P. (2024). Extracellular vesicles mediate inflammasome signaling in the brain and heart of alzheimer’s disease mice. Front. Mol. Neurosci..

[21] Liao J., Chen G., Liu X., Wei Z.Z., Yu S.P., Chen Q., Ye K. (2022). C/EBPβ/AEP signaling couples atherosclerosis to the pathogenesis of alzheimer’s disease. Mol. Psychiatry.

[22] Fazio S., Bellavite P., Affuso F. (2024). Chronically Increased Levels of Circulating Insulin Secondary to Insulin Resistance: A Silent Killer. Biomedicines.

[23] Vallee A., Vallee J.-N., Lecarpentier Y. (2022). WNT/β-catenin pathway: A possible link between hypertension and alzheimer’s disease. Curr. Hypertens. Rep..

[24] Patel V., Edison P. (2024). Cardiometabolic risk factors and neurodegeneration: A review of the mechanisms underlying diabetes, obesity and hypertension in Alzheimer’s disease. J. Neurol. Neurosurg. Psychiatry.

[25] Koskeridis F., Fancy N., Tan P.F., Meena D., Evangelou E., Elliott P., Wang D., Matthews P.M., Dehghan A., Tzoulaki I. (2024). Multi-trait association analysis reveals shared genetic loci between Alzheimer’s disease and cardiovascular traits. Nat. Commun..

[26] Chen Y., Jin H., Chen J., Li J., Găman M.A., Zou Z. (2025). The multifaceted roles of apolipoprotein E4 in Alzheimer’s disease pathology and potential therapeutic strategies. Cell Death Discov..

[27] Liu C., Liu J., Wang Y.-Y., Xu S.-F., Yu L.-M. (2025). APOE lipoprotein particles: Pathophysiology, therapy, and the crosstalk in alzheimer’s disease and cardiovascular disease. Mol. Neurobiol..

[28] Zhou Y., Yang P., He H., Liu H., Ye P., Xia J. (2026). DAB2IP: Unifying cardiovascular pathogenesis and cardiovascular brain crosstalk. Front. Cardiovasc. Med..

[29] Wang J., Yao J., Wang Z. (2025). Identification of shared mechanisms between alzheimer’s disease and atherosclerosis by integrated bioinformatics analysis. Eur. J. Med. Res..

[30] Agnello L., Giglio R.V., Del Ben F., Piccoli T., Colletti T., Scazzone C., Lo Sasso B., Ciaccio A.M., Gambino C.M., Salemi G. (2024). Evaluation of core Biomarkers of Alzheimer’s disease in saliva and plasma measured by chemiluminescent enzyme immunoassays on a fully automated platform. Sci. Rep..

[31] Agnello L., Gambino C.M., Ciaccio A.M., Cacciabaudo F., Massa D., Masucci A., Tamburello M., Vassallo R., Midiri M., Scazzone C. (2025). From Amyloid to Synaptic Dysfunction: Biomarker-Driven Insights into Alzheimer’s Disease. Curr. Issues Mol. Biol..

[32] Pradeepkiran J.A., Baig J., Islam M.A., Kshirsagar S., Reddy H. (2025). Amyloid-β and phosphorylated tau are the key biomarkers and predictors of alzheimer’s disease. Aging Dis..

[33] Ennis G.E., Norton D., Langhough R.E., Gooding D.C., Carter F.P., Wilson R., Zuelsdorff M., Bouges S., Asthana S., Johnson S.C. (2025). The performance of plasma p-tau217 in black middle-aged and older adults. Alzheimers Dement..

[34] French S.R., Arias J.C., Zahra S., Ally M., Escareno C., Heitkamp E., Vazquez F., Hillis M., Wiskoski H., Ainapurapu K. (2025). Cognitive impairment and p-tau217 are high in a vascular patient cohort. Alzheimers Dement..

[35] Rosenich E., Lim Y.Y. (2025). Ante-mortem cognitive trajectories associated with aβ and tau biomarker profiles in older adults with cerebrovascular disease: A longitudinal cohort study. Alzheimers Res. Ther..

[36] Cavalcante P.N., Kanhouche G., Rosa V.E.E., Campos C.M., Lopes M.P., Lopes M., Sampaio R.O., de Brito Júnior F.S., Tarasoutchi F., Abizaid A.A.C. (2023). B-type natriuretic peptide and N-terminal Pro-B-type natriuretic peptide in severe aortic stenosis: A comprehensive literature review. Front. Cardiovasc. Med..

[37] Diana G., Locorotondo G., Manfredonia L., Graziani F., Lombardo A., Lanza G.A., Pedicino D., Liuzzo G., Massetti M., Crea F. (2022). Subclinical dysfunction of remote myocardium is related to high NT-proBNP and affects global contractility at follow-up, independently of infarct area. Front. Cardiovasc. Med..

[38] Ungvari A., Nyúl-Tóth Á., Patai R., Csik B., Gulej R., Nagy D., Shanmugarama S., Benyó Z., Kiss T., Ungvari Z. (2025). Cerebromicrovascular senescence in vascular cognitive impairment: Does accelerated microvascular aging accompany atherosclerosis?. GeroScience.

[39] Habibi P., Chen L.Y., Sorond F.A., Shroff G.R., Sabayan B. (2026). Heart-brain axis: Subclinical cardiovascular changes and brain health. J. Am. Heart Assoc..

[40] Jensen M., Zeller T., Twerenbold R., Thomalla G. (2023). Circulating cardiac biomarkers, structural brain changes, and dementia: Emerging insights and perspectives. Alzheimers Dement..

[41] Ostovaneh M.R., Moazzami K., Yoneyama K., Venkatesh B.A., Heckbert S.R., Wu C.O., Shea S., Post W.S., Fitzpatrick A.L., Burke G.L. (2020). Change in N-terminal pro-B-type natriuretic peptide level and risk of dementia: Multi-ethnic study of atherosclerosis. Hypertension.

[42] Xiao T., van der Velpen I.F., Niessen W.J., Tilly M.J., Kavousi M., Ikram M.A., Ikram M.K., Vernooij M.W. (2023). NT-proBNP and changes in cognition and global brain structure: The rotterdam study. Eur. J. Neurol..

[43] Akamine S., Marutani N., Kanayama D., Gotoh S., Maruyama R., Yanagida K., Sakagami Y., Mori K., Adachi H., Kozawa J. (2020). Renal function is associated with blood neurofilament light chain level in older adults. Sci. Rep..

[44] Li Q., Su S., Feng Y., Jia M., Zhan J., Liao Z., Li J., Li X. (2024). Potential role of blood pressure variability and plasma neurofilament light in the mechanism of comorbidity between Alzheimer’s disease and cerebral small vessel disease. Alzheimers Dement..

[45] Bermudez C., Syrjanen J.A., Stricker N.H., Algeciras-Schimnich A., Kouri N., Kremers W.K., Petersen R.C., Jack C.R., Knopman D.S., Dickson D.W. (2025). Impact of cardiovascular risk factors on plasma biomarkers in prediction of Alzheimer’s and cerebrovascular neuropathology. J. Prev. Alzheimers Dis..

[46] Edwards N.C., Lao P., Alshikho M.J., Mazen J., Huber B., Hale C., Berroa J., Morel N., Pacheco A., Walker S. (2025). Alzheimer disease, vascular disease, and blood-brain barrier permeability biomarkers in middle-aged adults. Neurology.

[47] Lockhart S.N., Sutphen C.L., Tanley J., Gonzalez-Ortiz F., Kac P.R., Habes M., Heckbert S.R., Ashton N.J., Mielke M.M., Koeppe R. (2026). Plasma and neuroimaging biomarkers of small vessel disease and alzheimer’s disease in a diverse cohort: MESA. Alzheimers Dement..

[48] Dhana A., Decarli C.S., Dhana K., Desai P., Ng T.K.S., Evans D.A., Rajan K.B. (2025). Cardiovascular health and biomarkers of neurodegenerative disease in older adults. JAMA Netw. Open.

[49] Bridel C., van Wieringen W.N., Zetterberg H., Tijms B.M., Teunissen C.E., Group A.T.N., Alvarez-Cermeño J.C., Andreasson U., Axelsson M., Bäckström D.C. (2019). Diagnostic value of cerebrospinal fluid neurofilament light protein in neurology: A systematic review and meta-analysis. JAMA Neurol..

[50] Jung Y., Damoiseaux J.S. (2024). The potential of blood neurofilament light as a marker of neurodegeneration for Alzheimer’s disease. Brain.

[51] Middeldorp J., Hol E.M. (2011). GFAP in health and disease. Prog. Neurobiol..

[52] Messing A., Brenner M. (2020). GFAP at 50. ASN Neuro.

[53] Bellaver B., Povala G., Ferreira P.C.L., Ferrari-Souza J.P., Leffa D.T., Lussier F.Z., Benedet A.L., Ashton N.J., Triana-Baltzer G., Kolb H.C. (2023). Astrocyte reactivity influences amyloid-β effects on tau pathology in preclinical alzheimer’s disease. Nat. Med..

[54] Benedet A.L., Milà-Alomà M., Vrillon A., Ashton N.J., Pascoal T.A., Lussier F., Karikari T.K., Hourregue C., Cognat E., Dumurgier J. (2021). Differences between plasma and cerebrospinal fluid glial fibrillary acidic protein levels across the alzheimer disease continuum. JAMA Neurol..

[55] Pereira J.B., Janelidze S., Smith R., Mattsson-Carlgren N., Palmqvist S., Teunissen C.E., Zetterberg H., Stomrud E., Ashton N.J., Blennow K. (2021). Plasma GFAP is an early marker of amyloid-β but not tau pathology in alzheimer’s disease. Brain.

[56] Guillén N., Esteller D., Sarto J., Perales I., Ríos-Guillermo J., Massons M., Martín-Sobrino I., Augé J.M., Naranjo L., Ruiz-García R. (2025). Plasma p-Tau217 and GFAP predict widespread cognitive decline in Alzheimer’s disease. J. Neurol..

[57] Wu C.Y., Chen L., Fatima H., Gatchel J., Das S., Kivisäkk P., Arnold S.E., Dodge H.H. (2025). Combined use of plasma p-tau217, NfL, and GFAP predicts domain-specific cognitive decline in cognitively unimpaired and MCI individuals. Alzheimers Dement..

[58] Condrat C.E., Thompson D.C., Barbu M.G., Bugnar O.L., Boboc A., Cretoiu D., Suciu N., Cretoiu S.M., Voinea S.C. (2020). miRNAs as biomarkers in disease: Latest findings regarding their role in diagnosis and prognosis. Cells.

[59] Wiedrick J.T., Phillips J.I., Lusardi T.A., McFarland T.J., Lind B., Sandau U.S., Harrington C.A., Lapidus J.A., Galasko D.R., Quinn J.F. (2019). Validation of MicroRNA Biomarkers for Alzheimer’s Disease in Human Cerebrospinal Fluid. J. Alzheimers Dis..

[60] Corsten M.F., Dennert R., Jochems S., Kuznetsova T., Devaux Y., Hofstra L., Wagner D.R., Staessen J.A., Heymans S., Schroen B. (2010). Circulating MicroRNA-208b and MicroRNA-499 reflect myocardial damage in cardiovascular disease. Circ. Cardiovasc. Genet..

[61] Tijsen A.J., Creemers E.E., Moerland P.D., de Windt L.J., van der Wal A.C., Kok W.E., Pinto Y.M. (2010). MiR423-5p as a circulating biomarker for heart failure. Circ. Res..

[62] Perdoncin M., Konrad A., Wyner J.R., Lohana S., Pillai S.S., Pereira D.G., Lakhani H.V., Sodhi K. (2021). A review of miRNAs as biomarkers and effect of dietary modulation in obesity associated cognitive decline and neurodegenerative disorders. Front. Mol. Neurosci..

[63] Zhou M., Pang X.E. (2025). Polyphenols and miRNA interplay: A novel approach to combat apoptosis and inflammation in alzheimer’s disease. Front. Aging Neurosci..

[64] Huang J.P., Chang C.C., Kuo C.Y., Huang K.J., Sokal E.M., Chen K.H., Hung L.M. (2022). Exosomal microRNAs miR-30d-5p and miR-126a-5p Are Associated with Heart Failure with Preserved Ejection Fraction in STZ-Induced Type 1 Diabetic Rats. Int. J. Mol. Sci..

[65] Wang J., Han B., Zhao X., Sun Y., Liu Y., Hu F., Meng K. (2021). MicroRNA-532-5p upregulation protects neurological deficits after ischemic stroke through inhibition of BTB and CNC homology 1. Int. Immunopharmacol..

[66] Liu X., Li H., Hastings M.H., Xiao C., Damilano F., Platt C., Lerchenmüller C., Zhu H., Wei X.P., Yeri A. (2024). miR-222 inhibits pathological cardiac hypertrophy and heart failure. Cardiovasc. Res..

[67] Wan D.-F., Hao Z., Huang Y., Pan S.-S. (2022). Late exercise preconditioning regulates BNP increasing to assist the cardioprotection via up-regulation of NPR-a and down-regulation of NPR-C in rat myocardium. Int. J. Pept. Res. Ther..

[68] Santoso A., Maulana R., Alzahra F., Prameswari H.S., Ambari A.M., Hartopo A.B., Arso I.A., Radi B. (2020). The effects of aerobic exercise on N-terminal pro-B-type natriuretic peptide and cardiopulmonary function in patients with heart failure: A meta-analysis of randomised clinical trials. Heart Lung Circ..

[69] Sun Y., Huang X., Hu B., Wu Z., Zhang Y., Yuan Y., Feng L. (2025). The effects of early exercise on cardiovascular biomarkers in patients with congestive heart failure. ESC Heart Fail..

[70] Chistiakov D.A., Orekhov A.N., Bobryshev Y.V. (2016). The role of miR-126 in embryonic angiogenesis, adult vascular homeostasis, and vascular repair and its alterations in atherosclerotic disease. J. Mol. Cell. Cardiol..

[71] Marchegiani F., Recchioni R., Di Rosa M., Piacenza F., Marcheselli F., Bonfigli A.R., Galeazzi R., Matacchione G., Cardelli M., Procopio A.D. (2024). Low circulating levels of miR-17 and miR-126-3p are associated with increased mortality risk in geriatric hospitalized patients affected by cardiovascular multimorbidity. Geroscience.

[72] Ma Y., Liu H., Wang Y., Xuan J., Gao X., Ding H., Ma C., Chen Y., Yang Y. (2022). Roles of physical exercise-induced MiR-126 in cardiovascular health of type 2 diabetes. Diabetol. Metab. Syndr..

[73] Song W., Liang Q., Cai M., Tian Z. (2020). HIF-1α-induced up-regulation of microRNA-126 contributes to the effectiveness of exercise training on myocardial angiogenesis in myocardial infarction rats. J. Cell. Mol. Med..

[74] Riedel S., Radzanowski S., Bowen T.S., Werner S., Erbs S., Schuler G., Adams V. (2015). Exercise training improves high-density lipoprotein-mediated transcription of proangiogenic microRNA in endothelial cells. Eur. J. Prev. Cardiol..

[75] Wang H., Xie Y., Guan L., Elkin K., Xiao J. (2021). Targets identified from exercised heart: Killing multiple birds with one stone. npj Regen. Med..

[76] Liu X., Xiao J., Zhu H., Wei X., Platt C., Damilano F., Xiao C., Bezzerides V., Boström P., Che L. (2015). miR-222 is necessary for exercise-induced cardiac growth and protects against pathological cardiac remodeling. Cell Metab..

[77] Liu S., Li H., Shen Y., Zhu W., Wang Y., Wang J., Zhang N., Li C., Xie L., Wu Q. (2023). Moxibustion improves hypothalamus Aqp4 polarization in APP/PS1 mice: Evidence from spatial transcriptomics. Front. Aging Neurosci..

[78] VanPelt J., Page R.C. (2017). Unraveling the CHIP:Hsp70 complex as an information processor for protein quality control. Biochim. Biophys. Acta Proteins Proteom..

[79] Porchietto E., Morello G., Cicilese G., Rainero I., Rubino E., Tamagno E., Boschi S., Guglielmotto M. (2025). UCH-L1 in Alzheimer’s Disease: A Crucial Player in Dementia-Associated Mechanisms. Int. J. Mol. Sci..

[80] Xu L., Li M., Wei A., Yang M., Li C., Liu R., Zheng Y., Chen Y., Wang Z., Wang K. (2022). Treadmill exercise promotes E3 ubiquitin ligase to remove amyloid β and P-tau and improve cognitive ability in APP/PS1 transgenic mice. J. Neuroinflamm..

[81] Sipos É., Komoly S., Ács P. (2018). Quantitative Comparison of Primary Cilia Marker Expression and Length in the Mouse Brain. J. Mol. Neurosci..

[82] Singh M., Garrison J.E., Wang K., Sheffield V.C. (2019). Absence of BBSome function leads to astrocyte reactivity in the brain. Mol. Brain.

[83] Cao W., Lin J., Xiang W., Liu J., Wang B., Liao W., Jiang T. (2022). Physical exercise-induced astrocytic neuroprotection and cognitive improvement through primary cilia and mitogen-activated protein kinases pathway in rats with chronic cerebral hypoperfusion. Front. Aging Neurosci..

[84] Han H., Zhao Y., Du J., Wang S., Yang X., Li W., Song J., Zhang S., Zhang Z., Tan Y. (2023). Exercise improves cognitive dysfunction and neuroinflammation in mice through Histone H3 lactylation in microglia. Immun. Ageing.

[85] Roccati E., Collins J.M., Callisaya M.L., Alty J.E., King A.E., Brady J.J.R., Vickers J.C. (2025). Physical activity and blood-based biomarkers of neurodegeneration in community dwelling Australians from ISLAND (Island Study Linking Ageing and Neurodegenerative Disease). Alzheimers Dement..

[86] Wu X.-D., Zeng K., Liu W.-L., Gao Y.-G., Gong C.-S., Zhang C.-X., Chen Y.-Q. (2014). Effect of aerobic exercise on miRNA-TLR4 signaling in atherosclerosis. Int. J. Sports Med..

[87] Mayr B., Neudorfer M., Wurhofer D., Kilian C., Strumegger E.-M., Sareban M., Niebauer J. (2024). Effects of structured exercise training on miRNA expression in previously sedentary individuals. PLoS ONE.

[88] Witvrouwen I., Gevaert A.B., Possemiers N., Ectors B., Stoop T., Goovaerts I., Boeren E., Hens W., Beckers P.J., Vorlat A. (2021). Plasma-derived microRNAs are influenced by acute and chronic exercise in patients with heart failure with reduced ejection fraction. Front. Physiol..

[89] Sawada S., Kon M., Wada S., Ushida T., Suzuki K., Akimoto T. (2013). Profiling of circulating microRNAs after a bout of acute resistance exercise in humans. PLoS ONE.

[90] Kim S.A., Shin D., Ham H., Kim Y., Gu Y., Kim H.J., Na D.L., Zetterberg H., Blennow K., Seo S.W. (2025). Physical activity, alzheimer plasma biomarkers, and cognition. JAMA Netw. Open.

[91] Coulton J.B., He Y., Barthélemy N.R., Jiang H., Holtzman D.M., Bateman R.J. (2024). Multi-peptide characterization of plasma neurofilament light chain in preclinical and mild Alzheimer’s disease. Brain Commun..

[92] Liu J., Zhang Y. (2024). Serum neurofilament light chain: A novel biomarker for cardiovascular diseases in individuals without hypertension. Sci. Rep..

[93] Joisten N., Rademacher A., Warnke C., Proschinger S., Schenk A., Walzik D., Knoop A., Thevis M., Steffen F., Bittner S. (2021). Exercise diminishes plasma neurofilament light chain and reroutes the kynurenine pathway in multiple sclerosis. Neurol.-Neuroimmunol. Neuroinflammation.

[94] Chen X., Toueg T.N., Harrison T.M., Baker S.L., Jagust W.J. (2024). Regional Tau Deposition Reflects Different Pathways of Subsequent Neurodegeneration and Memory Decline in Cognitively Normal Older Adults. Ann. Neurol..

[95] Lin T.W., Shih Y.H., Chen S.J., Lien C.H., Chang C.Y., Huang T.Y., Chen S.H., Jen C.J., Kuo Y.M. (2015). Running exercise delays neurodegeneration in amygdala and hippocampus of Alzheimer’s disease (APP/PS1) transgenic mice. Neurobiol. Learn. Mem..

[96] Tarumi T., Patel N.R., Tomoto T., Pasha E., Khan A.M., Kostroske K., Riley J., Tinajero C.D., Wang C., Hynan L.S. (2022). Aerobic exercise training and neurocognitive function in cognitively normal older adults: A one-year randomized controlled trial. J. Intern. Med..

[97] Raji C.A., Meysami S., Hashemi S., Garg S., Akbari N., Ahmed G., Chodakiewitz Y.G., Nguyen T.D., Niotis K., Merrill D.A. (2024). Exercise-Related Physical Activity Relates to Brain Volumes in 10,125 Individuals. J. Alzheimers Dis..

[98] Shi H., Koronyo Y., Rentsendorj A., Regis G.C., Sheyn J., Fuchs D.T., Kramerov A.A., Ljubimov A.V., Dumitrascu O.M., Rodriguez A.R. (2020). Identification of early pericyte loss and vascular amyloidosis in Alzheimer’s disease retina. Acta Neuropathol..

[99] Liang X., Fa W., Wang N., Peng Y., Liu C., Zhu M., Tian N., Wang Y., Han X., Qiu C. (2023). Exosomal miR-532-5p induced by long-term exercise rescues blood-brain barrier function in 5XFAD mice via downregulation of EPHA4. Aging Cell.

[100] Henriksen E.J. (2002). Invited review: Effects of acute exercise and exercise training on insulin resistance. J. Appl. Physiol. (1985).

[101] Khaledi K., Hoseini R., Gharzi A. (2023). Effects of aerobic training and vitamin D supplementation on glycemic indices and adipose tissue gene expression in type 2 diabetic rats. Sci. Rep..

[102] Gu C., Yan J., Zhao L., Wu G., Wang Y.L. (2021). Regulation of Mitochondrial Dynamics by Aerobic Exercise in Cardiovascular Diseases. Front. Cardiovasc. Med..

[103] Craft S., Peskind E., Schwartz M.W., Schellenberg G.D., Raskind M., Porte D. (1998). Cerebrospinal fluid and plasma insulin levels in Alzheimer’s disease: Relationship to severity of dementia and apolipoprotein E genotype. Neurology.

[104] Brown C., Pemberton S., Babin A., Abdulhameed N., Noonan C., Brown M.B., Banks W.A., Rhea E.M. (2022). Insulin blood-brain barrier transport and interactions are greater following exercise in mice. J. Appl. Physiol..

[105] Villamil-Parra W., Moscoso-Loaiza L. (2024). Effects of physical exercise on Irisin and BDNF concentrations, and their relationship with cardiometabolic and mental health of individuals with Metabolic Syndrome: A Systematic Review. Exp. Gerontol..

[106] Alomari M.A., Khabour O.F., Alawneh K., Alzoubi K.H., Maikano A.B. (2020). The importance of physical fitness for the relationship of BDNF with obesity measures in young normal-weight adults. Heliyon.

[107] Yan B., Mei Z., Tang Y., Song H., Wu H., Jing Q., Zhang X., Yan C., Han Y. (2023). FGF21-FGFR1 controls mitochondrial homeostasis in cardiomyocytes by modulating the degradation of OPA1. Cell Death Dis..

[108] Sun H., Lin W., Tang Y., Tu H., Chen T., Zhou J., Wang D., Xu Q., Niu J., Dong W. (2023). Sustained remission of type 2 diabetes in rodents by centrally administered fibroblast growth factor 4. Cell Metab..

[109] Wang J., Meng X., Yang J., Tang Y., Zeng F., Wang Y., Chen Z., Chen D., Zou R., Liu W. (2025). Improvements in Exercise for Alzheimer’s Disease: Highlighting FGF21-Induced Cerebrovascular Protection. Neurochem. Res..

[110] Tomoto T., Tarumi T., Chen J.N., Hynan L.S., Cullum C.M., Zhang R. (2021). One-year aerobic exercise altered cerebral vasomotor reactivity in mild cognitive impairment. J. Appl. Physiol..

[111] Mendez Colmenares A., Voss M.W., Fanning J., Salerno E.A., Gothe N.P., Thomas M.L., McAuley E., Kramer A.F., Burzynska A.Z. (2021). White matter plasticity in healthy older adults: The effects of aerobic exercise. NeuroImage.

[112] Vidoni E.D., Morris J.K., Watts A., Perry M., Clutton J., Van Sciver A., Kamat A.S., Mahnken J., Hunt S.L., Townley R. (2021). Effect of aerobic exercise on amyloid accumulation in preclinical alzheimer’s: A 1-year randomized controlled trial. PLoS ONE.

[113] Burzynska A.Z., Chaddock-Heyman L., Voss M.W., Wong C.N., Gothe N.P., Olson E.A., Knecht A., Lewis A., Monti J.M., Cooke G.E. (2014). Physical activity and cardiorespiratory fitness are beneficial for white matter in low-fit older adults. PLoS ONE.

[114] Thompson P.D. (2005). Exercise prescription and proscription for patients with coronary artery disease. Circulation.

[115] Eston R., Connolly D. (1996). The use of ratings of perceived exertion for exercise prescription in patients receiving beta-blocker therapy. Sports Med..

[116] Banks N.F., Rogers E.M., Stanhewicz A.E., Whitaker K.M., Jenkins N.D.M. (2024). Resistance exercise lowers blood pressure and improves vascular endothelial function in individuals with elevated blood pressure or stage-1 hypertension. Am. J. Physiol. Heart Circ. Physiol..

[117] Kušleikienė S., Ziv G., Vints W.A.J., Krasinskė E., Šarkinaite M., Qipo O., Bautmans I., Himmelreich U., Masiulis N., Česnaitienė V.J. (2025). Cognitive gains and cortical thickness changes after 12 weeks of resistance training in older adults with low and high risk of mild cognitive impairment: Findings from a randomized controlled trial. Brain Res. Bull..

[118] Broadhouse K.M., Singh M.F., Suo C., Gates N., Wen W., Brodaty H., Jain N., Wilson G.C., Meiklejohn J., Singh N. (2020). Hippocampal plasticity underpins long-term cognitive gains from resistance exercise in MCI. NeuroImage Clin..

[119] Visseren F.L.J., Mach F., Smulders Y.M., Carballo D., Koskinas K.C., Bäck M., Benetos A., Biffi A., Boavida J.-M., Capodanno D. (2021). 2021 ESC guidelines on cardiovascular disease prevention in clinical practice. Eur. Heart J..

[120] Williams M.A., Haskell W.L., Ades P.A., Amsterdam E.A., Bittner V., Franklin B.A., Gulanick M., Laing S.T., Stewart K.J. (2007). Resistance exercise in individuals with and without cardiovascular disease: 2007 update: A scientific statement from the American Heart Association Council on Clinical Cardiology and Council on Nutrition, Physical Activity, and Metabolism. Circulation.

[121] Vona M., Codeluppi G.M., Iannino T., Ferrari E., Bogousslavsky J., von Segesser L.K. (2009). Effects of different types of exercise training followed by detraining on endothelium-dependent dilation in patients with recent myocardial infarction. Circulation.

[122] Dean A.S., Libonati J.R., Madonna D., Ratcliffe S.J., Margulies K.B. (2011). Resistance training improves vasoreactivity in end-stage heart failure patients on inotropic support. J. Cardiovasc. Nurs..

[123] Edelmann F., Wachter R., Duvinage A., Mueller S., Fegers-Wustrow I., Schwarz S., Christle J.W., Pieske-Kraigher E., Seyfarth M., Knapp M. (2025). Combined endurance and resistance exercise training in heart failure with preserved ejection fraction: A randomized controlled trial. Nat. Med..

[124] Montero-Odasso M., Zou G., Speechley M., Almeida Q.J., Liu-Ambrose T., Middleton L.E., Camicioli R., Bray N.W., Li K.Z.H., Fraser S. (2023). Effects of exercise alone or combined with cognitive training and vitamin D supplementation to improve cognition in adults with mild cognitive impairment: A randomized clinical trial. JAMA Netw. Open.

[125] Yu A.P., Chin E.C., Yu D.J., Fong D.Y., Cheng C.P., Hu X., Wei G.X., Siu P.M. (2022). Tai Chi versus conventional exercise for improving cognitive function in older adults: A pilot randomized controlled trial. Sci. Rep..

[126] Pedrinolla A., Venturelli M., Fonte C., Tamburin S., Di Baldassarre A., Naro F., Varalta V., Giuriato G., Ghinassi B., Muti E. (2020). Exercise training improves vascular function in patients with alzheimer’s disease. Eur. J. Appl. Physiol..

[127] Sanders L.M.J., Hortobágyi T., Karssemeijer E.G.A., Van der Zee E.A., Scherder E.J.A., van Heuvelen M.J.G. (2020). Effects of low- and high-intensity physical exercise on physical and cognitive function in older persons with dementia: A randomized controlled trial. Alzheimer’s Res. Ther..

[128] Li X., Chang P., Wu M., Jiang Y., Gao Y., Chen H., Tao L., Wei D., Yang X., Xiong X. (2024). Effect of tai chi vs aerobic exercise on blood pressure in patients with prehypertension: A randomized clinical trial. JAMA Netw. Open.

[129] Li Y., Li C., Wen J., Cui M., Wei Q., Liu M., Chen Z., Fang H., Liu L., Fu J. (2025). Tai chi as a mind-body exercise modulates endothelial function in coronary artery disease: A randomized clinical trial. Complement. Ther. Med..

[130] Zhou C. (2025). Effect of tai chi combined with music therapy on the cognitive function in older adult individuals with mild cognitive impairment. Front. Public Health.

[131] Li J., Yu M., Wang Y., Li S., Li S., Feng X., Li R., Chen K., Xu H. (2024). Baduanjin for ischemic heart failure with mildly reduced/preserved ejection fraction (BEAR trial): A randomized controlled trial. J. Evid.-Based Med..

[132] Prabhakaran D., Chandrasekaran A.M., Singh K., Mohan B., Chattopadhyay K., Chadha D.S., Negi P.C., Bhat P., Sadananda K.S., Ajay V.S. (2020). Yoga-based cardiac rehabilitation after acute myocardial infarction: A randomized trial. J. Am. Coll. Cardiol..

[133] Krause-Sorio B., Siddarth P., Kilpatrick L., Milillo M.M., Aguilar-Faustino Y., Ercoli L., Narr K.L., Khalsa D.S., Lavretsky H. (2022). Yoga prevents gray matter atrophy in women at risk for alzheimer’s disease: A randomized controlled trial. J. Alzheimer’s Dis. JAD.

[134] Hajjar I., Okafor M., Wan L., Yang Z., Nye J.A., Bohsali A., Shaw L.M., Levey A.I., Lah J.J., Calhoun V.D. (2022). Safety and biomarker effects of candesartan in non-hypertensive adults with prodromal Alzheimer’s disease. Brain Commun..

[135] Kawada K., Ishida T., Fukuda H., Hyohdoh Y., Kubo T., Hamada T., Baba Y., Hayashi T., Aizawa F., Yagi K. (2024). Effects of renin-angiotensin system inhibitor and beta-blocker use on mortality in older patients with heart failure with reduced ejection fraction in Japan. Front. Cardiovasc. Med..

[136] Ryan J., Storey E., Murray A.M., Woods R.L., Wolfe R., Reid C.M., Nelson M.R., Chong T.T.J., Williamson J.D., Ward S.A. (2020). Randomized placebo-controlled trial of the effects of aspirin on dementia and cognitive decline. Neurology.

[137] Nguyen T.N.M., Chen L.J., Trares K., Stocker H., Holleczek B., Beyreuther K., Brenner H., Schöttker B. (2022). Long-term low-dose acetylsalicylic use shows protective potential for the development of both vascular dementia and Alzheimer’s disease in patients with coronary heart disease but not in other individuals from the general population: Results from two large cohort studies. Alzheimers Res. Ther..

[138] Ridker P.M., Everett B.M., Thuren T., MacFadyen J.G., Chang W.H., Ballantyne C., Fonseca F., Nicolau J., Koenig W., Anker S.D. (2017). Antiinflammatory Therapy with Canakinumab for Atherosclerotic Disease. N. Engl. J. Med..

[139] Samuel M., Berry C., Dubé M.P., Koenig W., López-Sendón J., Maggioni A.P., Pinto F.J., Roubille F., Tardif J.C. (2025). Long-term trials of colchicine for secondary prevention of vascular events: A meta-analysis. Eur. Heart J..

[140] Andreotti F., Maggioni A.P., Campeggi A., Iervolino A., Scambia G., Massetti M. (2021). Anti-inflammatory therapy in ischaemic heart disease: From canakinumab to colchicine. Eur. Heart J. Suppl..

[141] Nidorf S.M., Fiolet A.T.L., Mosterd A., Eikelboom J.W., Schut A., Opstal T.S.J., The S.H.K., Xu X.F., Ireland M.A., Lenderink T. (2020). Colchicine in Patients with Chronic Coronary Disease. N. Engl. J. Med..

[142] Mehan S., Bhalla S., Siddiqui E.M., Sharma N., Shandilya A., Khan A. (2022). Potential Roles of Glucagon-Like Peptide-1 and Its Analogues in Dementia Targeting Impaired Insulin Secretion and Neurodegeneration. Degener. Neurol. Neuromuscul. Dis..

[143] Bach D.H., Nguyen T.L. (2026). Immunometabolism Reframes Alzheimer’s Disease: From Systemic Dysmetabolism to Glial Rewiring. Cell. Mol. Neurobiol..

[144] Evola V., Parmar M.S. (2026). Targeting neuroinflammation in neurodegenerative disorders: The emerging potential of semaglutide. Inflamm. Res..

[145] Ma L.Y., Liu S.F., Ma Z.Q., Guo Y.G., Li M., Gao Y., Wen Y.T., Niu Y., Sui H.X., Li B.S. (2025). Liraglutide improves cognition function in streptozotocin-induced diabetic rats by downregulating β-secretase and γ-secretase and alleviating oxidative stress in HT-22 cells. Endocr. J..

[146] Kostrzewska P., Kuca P., Witek P., Małyszko J., Madetko Alster N., Alster P. (2025). SGLT-2 Inhibitors in the Prevention and Progression of Neurodegenerative Diseases: A Narrative Review. Neurol. Ther..

[147] Song K., Choi J., Jeong D., Shin D., Ah Y.M., Lee K.Y., Choi K.H. (2025). Comparative effects of SGLT2 inhibitors and incretin-based therapies on dementia risk in type 2 diabetes: A systematic review and meta-analysis. Front. Endocrinol..

[148] van de Rest O., Berendsen A.A., Haveman-Nies A., de Groot L.C. (2015). Dietary patterns, cognitive decline, and dementia: A systematic review. Adv. Nutr..

[149] Mrhar A., Carballo-Casla A., Grande G., Valletta M., Fredolini C., Fratiglioni L., Gregorič Kramberger M., Kuhar A., Winblad B., Calderón-Larrañaga A. (2025). Dietary patterns and blood-based biomarkers of Alzheimer’s disease in cognitively intact older adults: Findings from a population-based study. J. Prev. Alzheimers Dis..

[150] Won S., Sayeed I., Peterson B.L., Wali B., Kahn J.S., Stein D.G. (2015). Vitamin D prevents hypoxia/reoxygenation-induced blood-brain barrier disruption via vitamin D receptor-mediated NF-kB signaling pathways. PLoS ONE.

[151] Arrol S., Mackness M.I., Durrington P.N. (2000). Vitamin E supplementation increases the resistance of both LDL and HDL to oxidation and increases cholesteryl ester transfer activity. Atherosclerosis.

[152] Puranik N., Kumari M., Tiwari S., Dhakal T., Song M. (2025). Resveratrol as a Therapeutic Agent in Alzheimer’s Disease: Evidence from Clinical Studies. Nutrients.

[153] Yang F., Lim G.P., Begum A.N., Ubeda O.J., Simmons M.R., Ambegaokar S.S., Chen P.P., Kayed R., Glabe C.G., Frautschy S.A. (2005). Curcumin inhibits formation of amyloid beta oligomers and fibrils, binds plaques, and reduces amyloid in vivo. J. Biol. Chem..

[154] Zhou B., Hu B. (2025). Anti-inflammatory effect of curcumin on neurological disorders: A narrative review. Front. Pharmacol..

[155] Ngandu T., Lehtisalo J., Solomon A., Levälahti E., Ahtiluoto S., Antikainen R., Bäckman L., Hänninen T., Jula A., Laatikainen T. (2015). A 2 year multidomain intervention of diet, exercise, cognitive training, and vascular risk monitoring versus control to prevent cognitive decline in at-risk elderly people (FINGER): A randomised controlled trial. Lancet.

[156] Olumuyide E., Ariel K., Aneni E. (2026). Integrating metabolic rehabilitation with incretin-based anti-obesity therapy: A narrative review of a multimodal strategy for sustainable weight loss. Int. J. Obes..

[157] Gliemann L., Schmidt J.F., Olesen J., Biensø R.S., Peronard S.L., Grandjean S.U., Mortensen S.P., Nyberg M., Bangsbo J., Pilegaard H. (2013). Resveratrol blunts the positive effects of exercise training on cardiovascular health in aged men. J. Physiol..

[158] Klonizakis M., Mitropoulos A. (2023). Assessing the effect of regular swimming exercise on the micro- and macrovascular physiology of older adults (ACELA II study). Front. Physiol..

[159] Blumenthal J.A., Smith P.J., Mabe S., Hinderliter A., Welsh-Bohmer K., Browndyke J.N., Doraiswamy P.M., Lin P.-H., Kraus W.E., Burke J.R. (2020). Longer term effects of diet and exercise on neurocognition: 1-year follow-up of the ENLIGHTEN trial. J. Am. Geriatr. Soc..

[160] Huang Y., Ou H., Zhao W., Lin Q., Xue Y., Xia R., Tan Z., Zhao X., Xiong L., Yan Z. (2024). The effects of moderate-intensity aerobic exercise on cognitive function in individuals with stroke-induced mild cognitive impairment: A randomized controlled pilot study. J. Rehabil. Med..

[161] Gaitán J.M., Moon H.Y., Stremlau M., Dubal D.B., Cook D.B., Okonkwo O.C., van Praag H. (2021). Effects of aerobic exercise training on systemic biomarkers and cognition in late middle-aged adults at risk for alzheimer’s disease. Front. Endocrinol..

[162] Souza W.M.M.d., Vieira M.C., Nascimento P.M.C., Serra S.M., Reis M.S. (2024). Strength training improves functional capacity of individuals with chronic heart failure: Randomized clinical trial. J. Bodyw. Mov. Ther..

[163] Reed J.L., Terada T., Vidal-Almela S., Tulloch H.E., Mistura M., Birnie D.H., Wells G.A., Nair G.M., Hans H., Way K.L. (2022). Effect of high-intensity interval training in patients with atrial fibrillation: A randomized clinical trial. JAMA Netw. Open.

